# Supplementary material for: Fate mapping of hematopoietic stem cells reveals two pathways of native thrombopoiesis
Source: Nat Commun. 2022 Aug 3;13:4504. doi: 10.1038/s41467-022-31914-z (PMC9349191; doi:10.1038/s41467-022-31914-z)
Supplement: Supplementary file 1 — Supplementary Information [file 41467_2022_31914_MOESM1_ESM.pdf]

Supplementary Figure 1

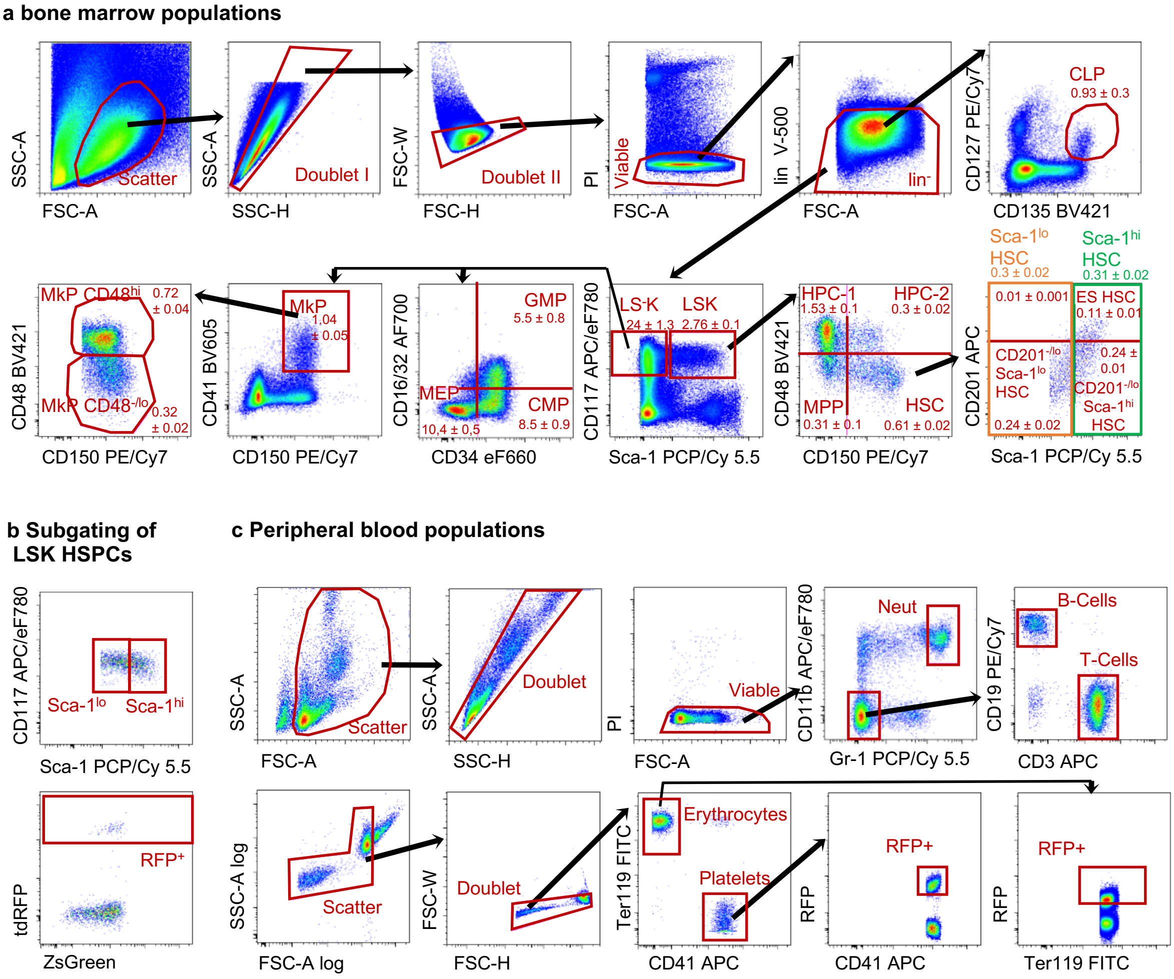

Supplementary Figure 1. Identification of bone marrow and peripheral blood populations.

a, Representative example of flow cytometry gating for bone marrow HSCs and progenitors; mean frequencies of populations ± SEM (n=4 mice) among lineage negative cells (lin^{-}) are shown (*LS^{-}K*, lin^{-} Sca-1^{-} CD117^{+} cells; *LSK*, lin^{-} Sca-1^{+} CD117^{+} cells; *common lymphoid progenitor (CLP),* lin^{-} CD135^{+} CD127^{+}; *megakaryocyte progenitor (MkP),* LS^{-}K CD41^{+} CD150^{+}; *granulocyte-macrophage progenitor (GMP),* LS^{-}K CD16/32^{+} CD34^{+}; *common myeloid progenitor (CMP),* LS^{-}K CD16/32^{-} CD34^{+}; *megakaryocyte-erythrocyte progenitor (MEP),* LS^{-}K CD16/32^{-} CD34^{-}; *restricted hematopoietic progenitor 1 (HPC-1),* LSK CD48^{hi} CD150^{-}; *restricted hematopoietic progenitor 2 (HPC-2),* LSK CD48^{hi} CD150^{+}; *multipotent progenitor (MPP),* LSK CD48$^{-/lo}$ CD150^{-}; *hematopoietic stem cell (HSC),* LSK CD48$^{-/lo}$ CD150^{+}; *ES HSC,* CD201^{hi} Sca-1^{hi} LSK CD48$^{-/lo}$ CD150^{+}; *CD201$^{-/lo}$ Sca-1^{hi} HSC,* CD201$^{-/lo}$ Sca-1^{hi} LSK CD48$^{-/lo}$ CD150^{+}; *Sca-1^{hi} HSC,* Sca-1^{hi} LSK CD48$^{-/lo}$ CD150^{+}; *CD201$^{-/lo}$ Sca-1^{lo} HSC,* CD201$^{-/lo}$ Sca-1^{lo} LSK CD48$^{-/lo}$ CD150^{+}). The RFP-labeling frequencies of induced *Fgd5*$^{ZsGreen:CreERT2}$/*R26*$^{LSL-tdRFP}$ mice were identified employing two independent staining panels (panel 1: HSC, MPP, HPC-1 and MkP, panel 2: CMP, GMP, MEP and CLP). Our analysis of un-related flow cytometry data encompassing all markers in a single antibody panel revealed that few events could be assigned to more than a single population. E.g. MkP and CMP as well as CLP and HPC-1 showed some minor overlap (<10% of events).

b, Representative sub-gating of LSK populations according to Sca-1 expression level (left). Representative gating of RFP^{+} bone marrow populations (right).

c, Representative gating of peripheral blood leukocytes (upper row, *neutrophilic granulocytes (Neut),* CD11b^{+} Gr-1^{+}; *B-Cells,* CD11b^{-} Gr-1^{-} CD19^{+}; *T-Cells,* CD11b^{-} Gr-1^{-} CD3^{+}) and erythrocytes and platelets (lower row, *erythrocytes,* CD41^{-} Ter119^{+}; *platelets,* CD41^{+} Ter119^{-}).

Supplementary Figure 2

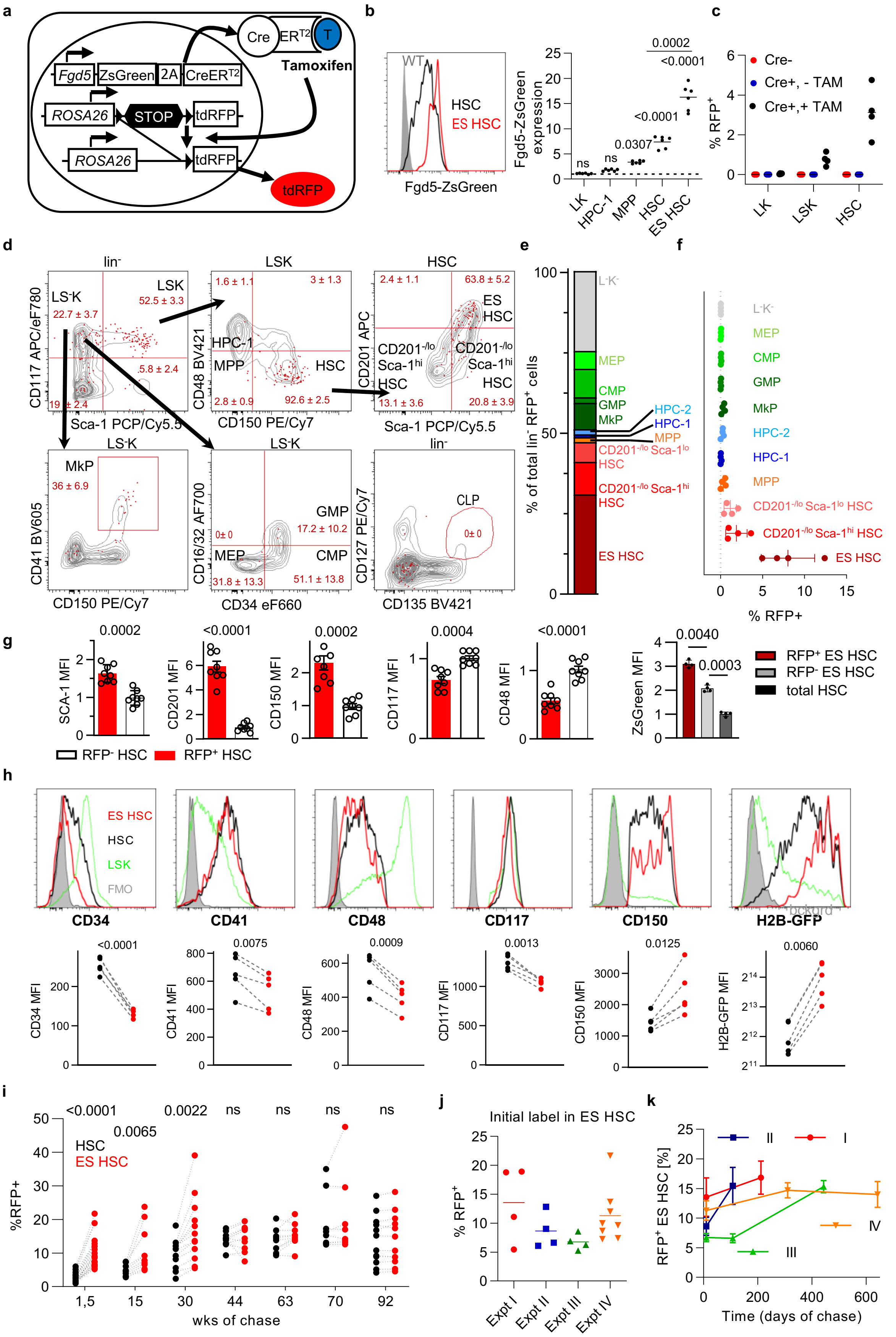

Supplementary Figure 2. ***Fgd5*$^{ZsGreen:CreERT2}$/*R26*$^{LSL\text{-}tdRFP}$ mouse model for selective labeling of HSCs.**

a, Schematic of the *Fgd5*$^{ZsGreen:CreERT2}$/*R26*$^{LSL\text{-}tdRFP}$ mouse model. The endogenous *Fgd5* promoter drives expression of a green fluorescent protein (ZsGreen) and tamoxifen-inducible Cre-estrogen receptor fusion protein (CreER^{T2}). TAM induction causes irreversible excision of the lox*P*-flanked stop cassette (LSL) from the *R26*$^{LSL\text{-}tdRFP}$ allele. Cre-recombined cells and their progeny are labeled by stable RFP expression.

b, ES HSCs (representative histogram shown in red) from *Fgd5*$^{ZsGreen:CreERT2}$*R26*$^{LSL\text{-}tdRFP}$ animals express higher levels of Fgd5-ZsGreen than the total HSC (black histogram, autofluorescence control shown in grey) population (two-tailed paired Student´s t test). Median Fgd5-ZsGreen fluorescence of bone marrow populations isolated from *Fgd5*$^{ZsGreen:CreERT2}$/*R26*$^{LSL\text{-}tdRFP}$ animals (n=6 mice, mean ± SEM) was normalized to the autofluorescence of the respective population (dotted line, *Fgd5*$^{wt/wt}$/*R26*$^{LSL\text{-}tdRFP}$ mice, n=3 mice). Significant Fgd5-ZsGreen expression was determined by an unpaired one-way ANOVA with Sidak error correction (ns, not significant; p values shown in graph).

c, *Fgd5*$^{ZsGreen:CreERT2}$/*R26*$^{LSL\text{-}tdRFP}$ mice (blue, n=3 mice, age 52 - 57 weeks, Cre+, -TAM) were deliberately left un-induced and potential leaky RFP activation was excluded. TAM-induced *Fgd5*$^{ZsGreen:CreERT2}$/*R26*$^{LSL\text{-}tdRFP}$ (black, n=4 mice, Cre+, +TAM) and Cre-negative (red, n=4 mice, Cre-) mice served as positive and negative controls, respectively.

d-f, *Fgd5*$^{ZsGreen:CreERT2}$/*R26*$^{LSL\text{-}tdRFP}$ mice (n=4 mice) were TAM-induced and bone marrow was analysed 10 days later. **d**, RFP^{+} lin^{-} cells (red dots) were overlaid to total lin^{-} cells (grey contour plots, representative animal; % ± SEM of RFP^{+} cells within each parental population is shown). **e**, RFP^{+} and total bone marrow populations were gated and displayed accordingly. The mean distribution of hematopoietic populations within total RFP^{+} lin^{-} bone marrow cells is shown. **f**, The mean frequency of RFP^{+} cells among the respective total populations is depicted. This revealed only sporadic labelling of non-HSCs and might even reflect continuous differentiation of labelled HSCs within the first days after labelling

g, *Fgd5*$^{ZsGreen:CreERT2}$/*R26*$^{LSL\text{-}tdRFP}$ mice (n=8 mice) were TAM-induced and bone marrow was analysed 10 days later. Expression (MFI, mean + SEM) of cell surface antigen of RFP^{+} (red bars) and RFP^{-} (open bars) HSCs (LSK CD48$^{-/lo}$ CD150^{+}) was normalized to the mean MFI of the total HSC population. Fgd5 reporter expression (right plot) was normalized and is shown for RFP^{+} (dark red) and RFP^{-} (grey) ES HSC in comparison to total HSC (black). Significances were calculated by a two-tailed paired Student´s t test; ns, not significant; p values shown in graph.

Supplementary Figure 2. *Fgd5*$^{ZsGreen:CreERT2}$*/R26*$^{LSL\text{-}tdRFP}$ mouse model for selective labeling of HSCs (continued).

h, Marker expression levels of total HSCs (black) and ES HSC (red) isolated from *R26*^{rtTA}*Col1A1*$^{H2B\text{-}GFP}$ mice (n=5 mice), which were pulsed with Dox for 5 weeks and chased for 11 weeks. Representative histograms are depicted (upper row). Mean fluorescence intensities (MFI) of paired (dotted line) HSCs and ES HSCs from each individual are shown (lower row, two-tailed paired Student´s t test; ns, not significant; p values shown in graph).

i, *Fgd5*$^{ZsGreen:CreERT2}$*/R26*$^{LSL\text{-}tdRFP}$ mice (n=74 mice, same as in Fig. 1c-e) were TAM-induced and ES HSCs (red) and HSCs (black) were analyzed between 10 to 642 days after induction for RFP expression. The labelling frequencies of ES HSCs and HSCs are shown pairwise for each individual (dotted line, two-tailed paired Student´s t test; ns, not significant; p values shown in graph).

j, *Fgd5*$^{ZsGreen:CreERT2}$*/R26*$^{LSL\text{-}tdRFP}$ mice (n=20 mice , same as Fig. 1b) were TAM-induced (4 independent experiments (Expt I-IV)) and ES HSCs were analyzed 10 - 11 days after TAM induction for RFP expression. Means and individual mice are shown.

k, *Fgd5*$^{ZsGreen:CreERT2}$*/R26*$^{LSL\text{-}tdRFP}$ mice (n=74 mice, same as in Fig. 1c-e) were TAM-induced (4 independent experiments I-IV) and ES HSCs were analyzed 10 - 642 days after TAM induction for RFP expression. The time course of RFP labeling in ES HSCs for each independent experiments is shown as mean values ± SEM.

Supplementary Figure 3

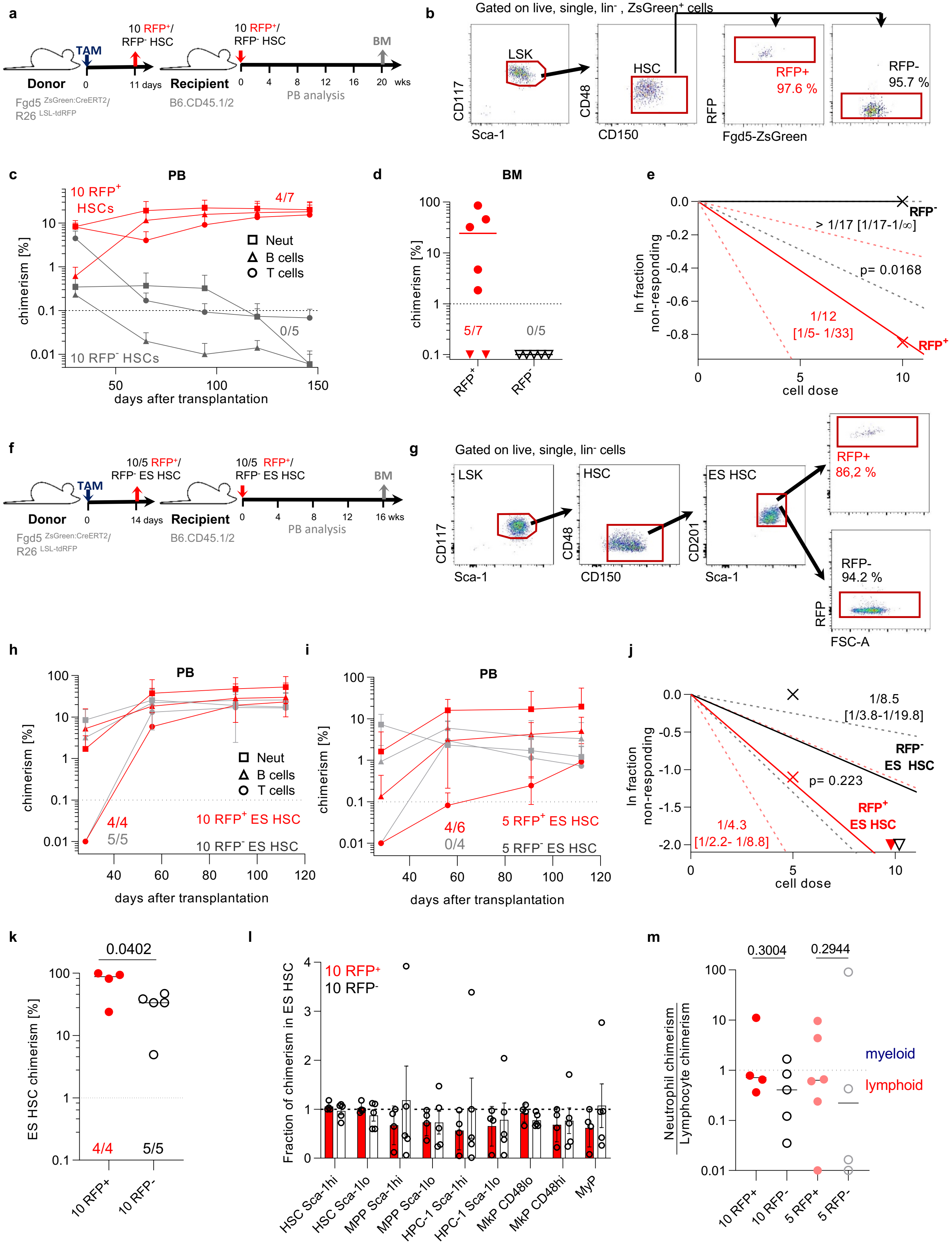

Supplementary Figure 3. Transplantation potential of different labelled and unlabeled HSC populations.

a-e, *Fgd5*$^{ZsGreen:CreERT2}$/*R26*$^{LSL\text{-}tdRFP}$ mice were TAM induced and 11 days later either 10 RFP^{-} (grey) or RFP^{+} (red) HSCs (LSK $CD48^{-/lo}$ $CD150^{+}$ $ZsGreen^{+}$) were competitively transplanted into irradiated recipient mice (n = 5 and 7 mice, respectively).

b, Re-analysis of sorted RFP^{+} and RFP^{-} donor HSCs. Purity of the respective sorted populations are shown.

c, Peripheral blood chimerism (mean and SEM are shown) was analysed and fractions of recipients with successful long-term multi-lineage reconstitution (< 0.1% chimerism (dotted line) in each lineage 146 days after transplantation) are shown.

d, Bone marrow populations were analyzed 147 days after transplantation for donor-derived cells (individual recipients and means are shown, mice without detectable chimerism are represented by triangles). Fractions of reconstituted mice (>1% chimerism in HSCs (dotted line)) are given.

e, j, Frequencies of functional HSCs with long-term multi-lineage (> 0.1 % contribution to neutrophils, B and T cells each at final PB analysis) repopulation activity among transplanted HSCs (e) or ES HSCs (j) were estimated by limiting dilution analysis. Frequencies, 95% confidence intervals (dotted lines and square brackets) and significance were calculated by ELDA (Hu and Smyth 2009).

f-m, *Fgd5*$^{ZsGreen:CreERT2}$/*R26*$^{LSL\text{-}tdRFP}$ mice were TAM induced and 14 days later either 10 or 5 RFP^{-} (grey) or RFP^{+} (red) ES HSCs (LSK $CD48^{-/lo}$ $CD150^{+}$ $CD201^{hi}$ Sca-1^{hi}) were competitively transplanted into irradiated recipient mice (n=5, 4, 4 and 6 mice, respectively).

g, Re-analysis of purified RFP^{+} and RFP^{-} donor ES HSCs. Purity of the respective sorted populations are shown.

h, i, Peripheral blood chimerism (mean and SEM are shown) of animals transplanted with 10 (h) or 5 (i) ES HSC was analysed and fractions of recipients with successful long-term multi-lineage reconstitution (< 0.1% chimerism (dotted line) in each PB lineage 112 days after transplantation) are shown.

Supplementary Figure 3. Transplantation potential of different labelled and unlabeled HSC populations (continued).

k, Bone marrow populations were analyzed 112 days after transplantation for donor-derived cells (individual recipients and means are shown, two-tailed unpaired Student´s t test; ns, not significant; p values shown in graph). Fractions of reconstituted mice (>1% chimerism in ES HSCs (dotted line)) are given.

l, Bone marrow repopulation pattern of recipient transplanted with 10 ES HSCs. The chimerism detected in each HSPC population was normalized to ES HSC chimerism of the same individual (mean and SEM are shown).

m, Lineage bias of recipient mice transplanted with ES HSC was calculated as the ratio of neutrophil and lymphoid chimerism in peripheral blood in the same individual (individual recipients and means are shown; two-tailed student’s t-Test, p-values are given in Figure).

Supplementary Figure 4

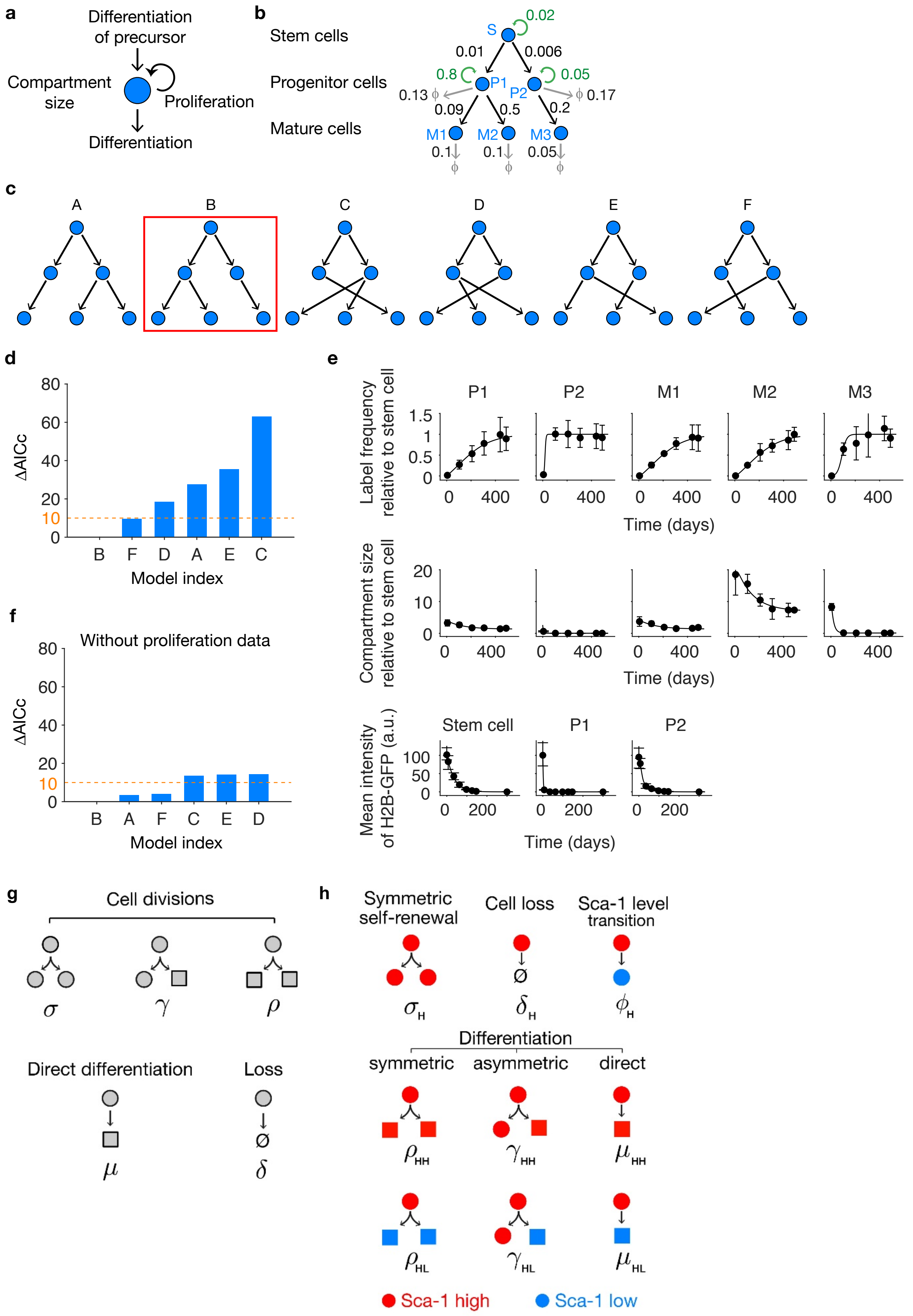

Supplementary Figure 4. Combining data on HSC fate-mapping and mitotic history allows inference of lineage pathways.

a, The number of cells in a given population is controlled by the rates of cell differentiation of this population and its precursors and the rate of cell proliferation of this population; in addition, cell death may also occur (not shown).

b, A generic lineage tree with a stem cell population (S) giving rise to two progenitors (P1 and P2), with P1 yielding mature cell types M1 and M2, and P2 yielding mature cell type M3. To simulate data on cell numbers, fate mapping from S and mitotic history (via H2B-GFP dilution), reasonable values are assigned to cell differentiation rates (black arrows), proliferation rates (green arrows) and death rates (grey arrows), with unit day^{-1}.

c, All possible topologies of lineage pathways connecting S to P1 and P2, and further to M1 through M3. For simplicity, convergence (P1 and P2 spawning the same mature cell type) has not been considered.

d, Model selection by evaluating how well the schemes a-f in (c) can account for the simulated fate-mapping, H2B-GFP dilution and cell number data computed with the model b. Ranking via the bias-corrected Akaike information criterion (AICc) shows that the original model (b) accounts significantly better for the data than any other model (ΔAICc of the remaining models all > 2).

e, The best fit of model b (curves) to the simulated data (n = 20; mean and SEM are shown).

f, Model selection using only the simulated fate-mapping data.

g, Illustration of fundamental cell fates of a stem or progenitor cell based on which the generic population dynamics equations (Equation 1 in the supplemental method) are derived.

h, Extended schemes when Sca-1^{hi} (red) and Sca-1^{lo} (blue) subpopulations are considered. Here the elementary processes of a Sca-1^{hi} stem/progenitor cell are considered, and they are symmetric to the Sca-1^{lo} cell. The subscripts H and L of the parameters stand for Sca-1^{hi} and Sca-1^{lo}, respectively. HH and HL represent the differentiation without and with the loss in Sca-1 level.

Supplementary Figure 5

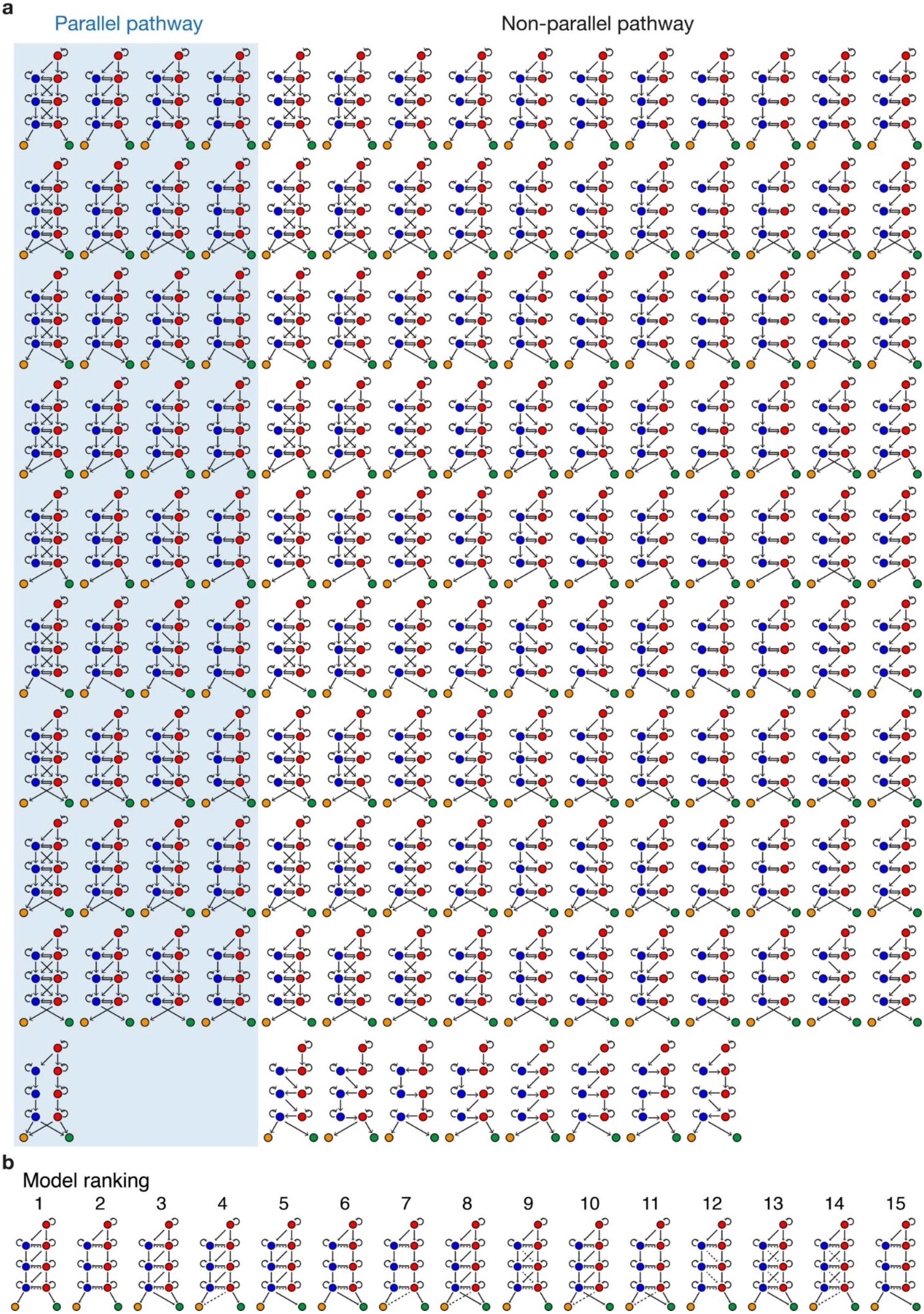

Supplementary Figure 5 (continued)

c

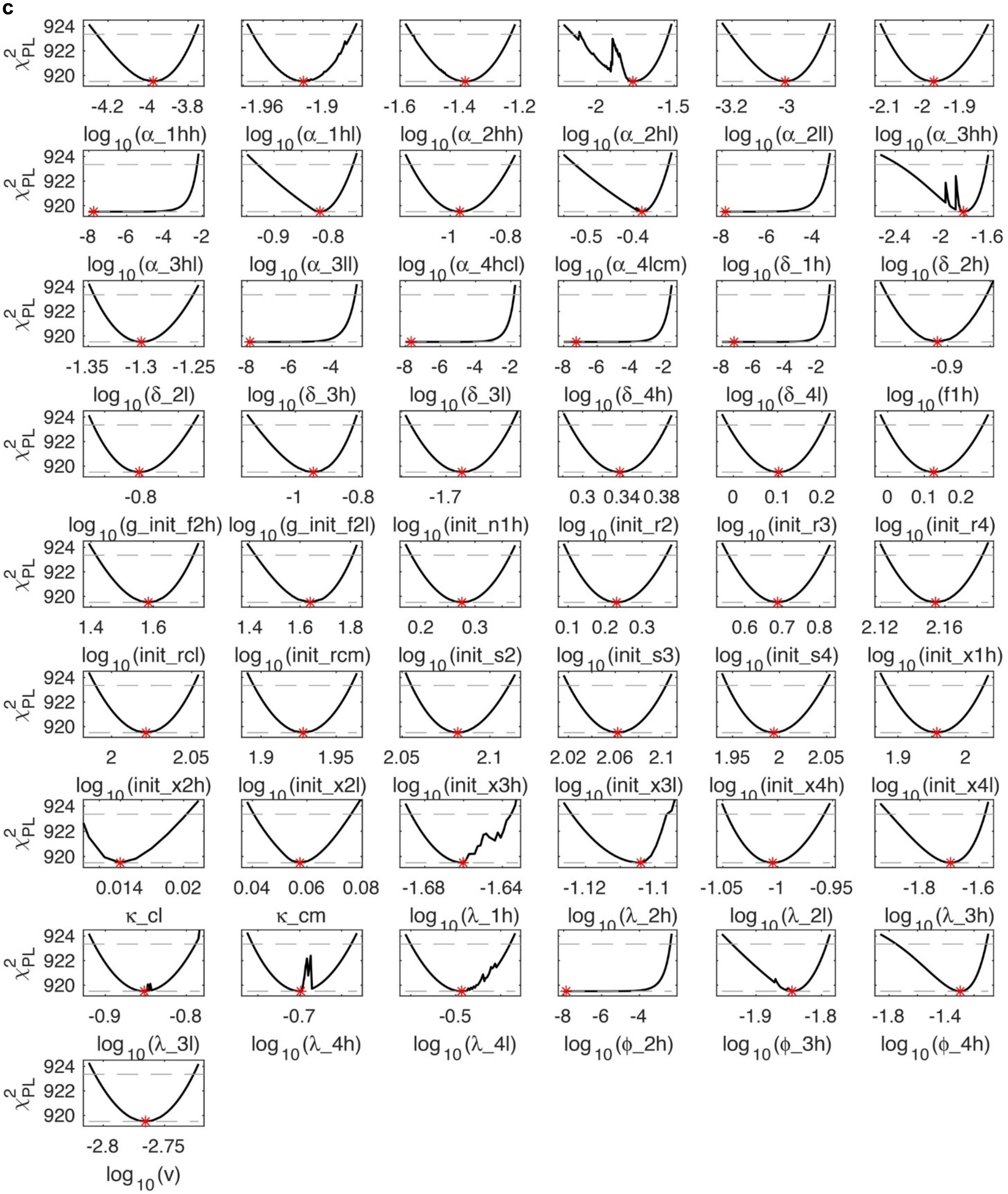

Supplementary Figure 5. Families of model schemes with different topologies of lineage pathways.

a, 144 models for statistical model selection. Schemes with parallel Sca-1^{hi} and Sca-1^{lo} pathways are shaded in blue. **b**, Top ranked models with ΔAICc < 10. **c,** Profile likelihood of all parameters of the best selected model (Model 1 in **b**).

Supplementary Figure 6

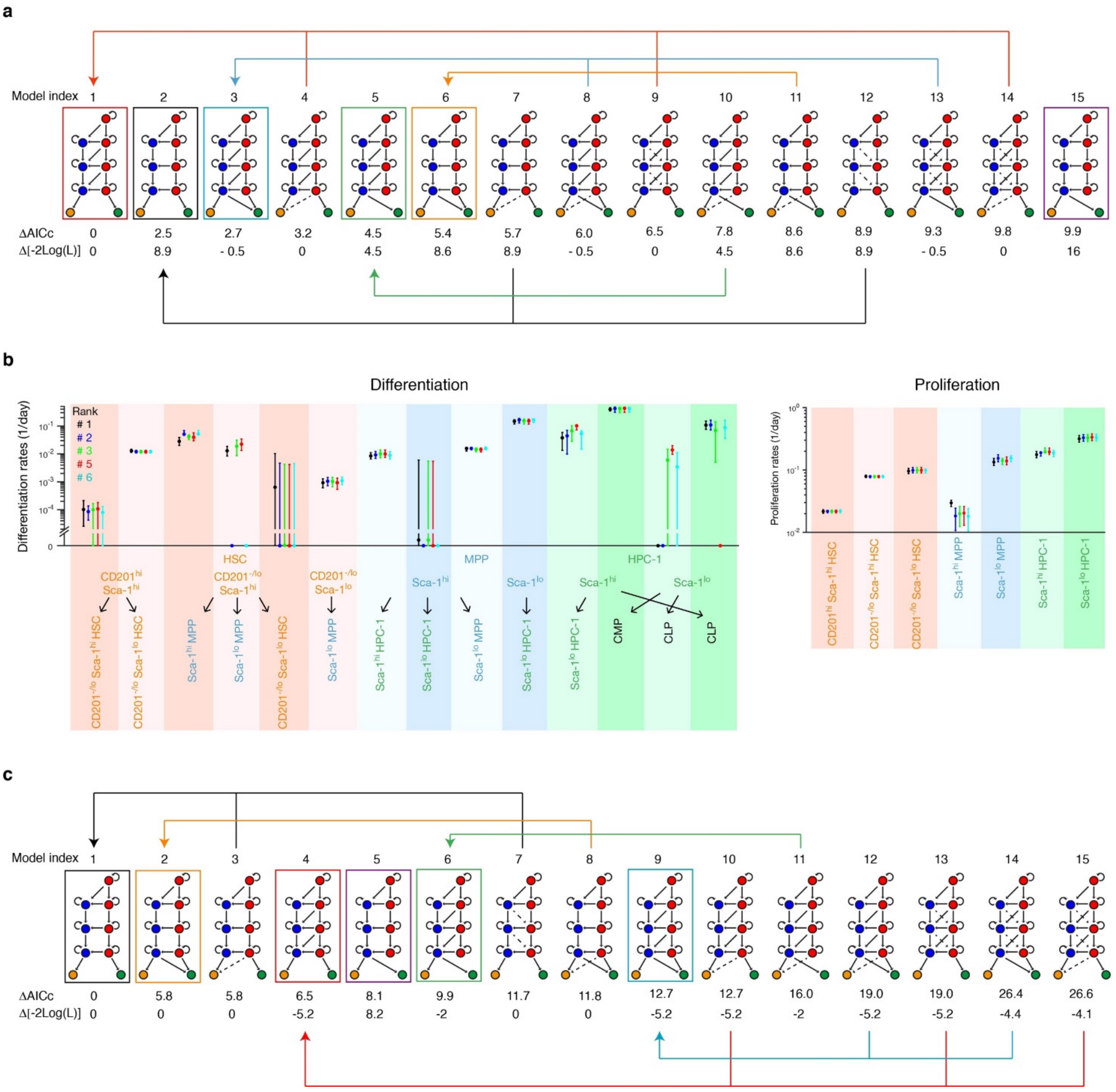

Supplementary Figure 6. Comparison among highly ranked models (with ΔAICc < 10).

a, The Akaike information criterion is defined as the likelihood of the data fitting penalized by the number of model parameters. Several models with complex topologies share the same likelihood values with their simple correspondence, and the higher ΔAICc values are solely due to extra number of parameters for certain transitions, marked by dashed lines. However, these extra parameters are inferred to be zeros (zero also being the open lower confidence bound). Therefore, 15 highly ranked models converge to only 6 core models (1, 2, 3, 5, 6 and 15). Since the Model 15 has a very bad score, it is excluded for further analyses. Δ[-2Log(Likelihood)] is the difference in -2Log(Likelihood) value with respect to Model 1.

b, The inferred differentiation (left panel) and proliferation (right panel) rates of the 5 core models, with 95% confidence bounds.

c, Model ranking inferred from using a partial data set (created by dropping every other time point). Model selection was performed among the original top 15 ranking models. Model reduction due to inferred zero transition rates also occur, and most core model topologies (in colored rectangles) remain selected (ΔAICc < 10). While the original best model in (**a**) ranks the 4th here, it still holds the highest likelihood (i.e., lowest Δ[-2Log(Likelihood)]).

Supplementary Figure 7

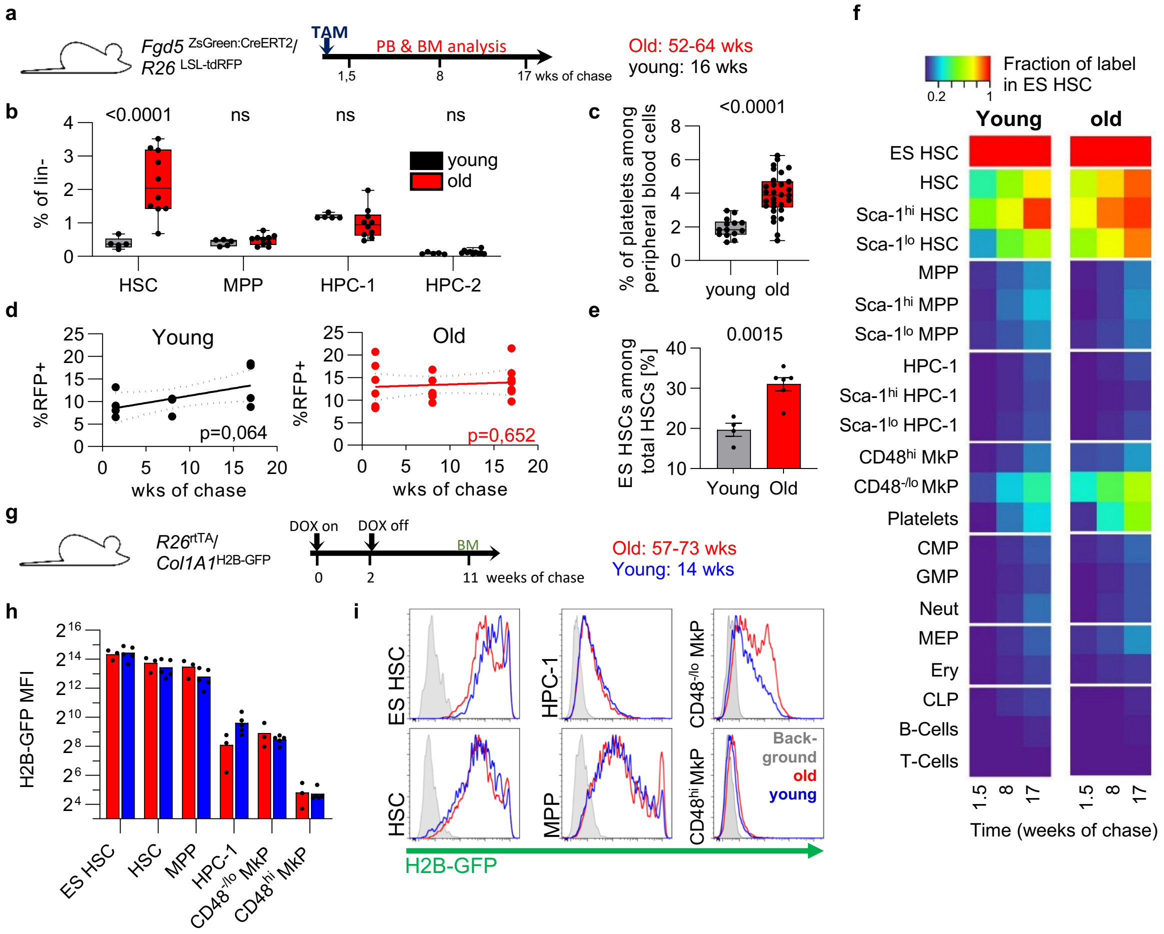

Supplementary Figure 7. Fate mapping in aged and young animals reveals changed dynamics upon ageing. (Part I)

a- f, Young (n=15) and old (n=30) *Fgd5*$^{ZsGreen:CreERT2}$/*R26*$^{LSL-tdRFP}$ mice were TAM- induced, groups of mice were sacrificed at indicated time points, and bone marrow and peripheral blood were analysed for RFP label propagation.

b, Frequency of HSC, MPP, HPC-1 and HPC-2 within total lineage^{-} cells after 120 days of chase in young (black, n=5) and old (red, n=10) animals. (box plots span 25th-75th percentile, medians are shown, whiskers extend to minimum and maximum value; unpaired one-way ANOVA with Sidak error correction; ns, not significant; p values shown in graph).

c, Frequency of platelets among all non-erylysed peripheral blood cells measured after 9 days of chase in young (black, n=14 mice) and old (red, n=30 mice) animals (box plots span 25th-75th percentile, medians are shown, whiskers extend to minimum and maximum value; , two-tailed unpaired Student´s t test; p value shown in graph).

d, Percentage of RFP^{+} ES HSCs in young (black) and old (red) animals. RFP-labeling of ES HSCs did not significantly rise (F-test on linear regression, p values shown in graph) in young or old animals.

e, Frequency of ES HSC within the total HSC compartment after 10 days of chase in young (black, n=4) and old (red, n=6) animals (bars represent means, SEM is shown).

f, Fractions of RFP-labeled peripheral blood and bone marrow cells relative to ES HSCs at indicated time points (normalised as in Figure 1e).

g- i, Young (n= 5) and old (n= 3) *R26*^{rtTA}/*Col1A1*$^{H2B-GFP}$ animals were Dox-induced and BM was analysed after 11 weeks of chase.

h, H2B-GFP mean fluorescence intensities (MFI) from individual mice and means thereof (bars) are shown in $\log_2$ display. Population-specific background H2B-GFP fluorescence was subtracted.

i, H2B-GFP histograms BM populations from representative old (red) and young (blue) *R26*^{rtTA}/*Col1A1*$^{H2B-GFP}$ animals. Background fluorescence of each population is depicted (grey histograms).

Supplementary Figure 7 (continued)

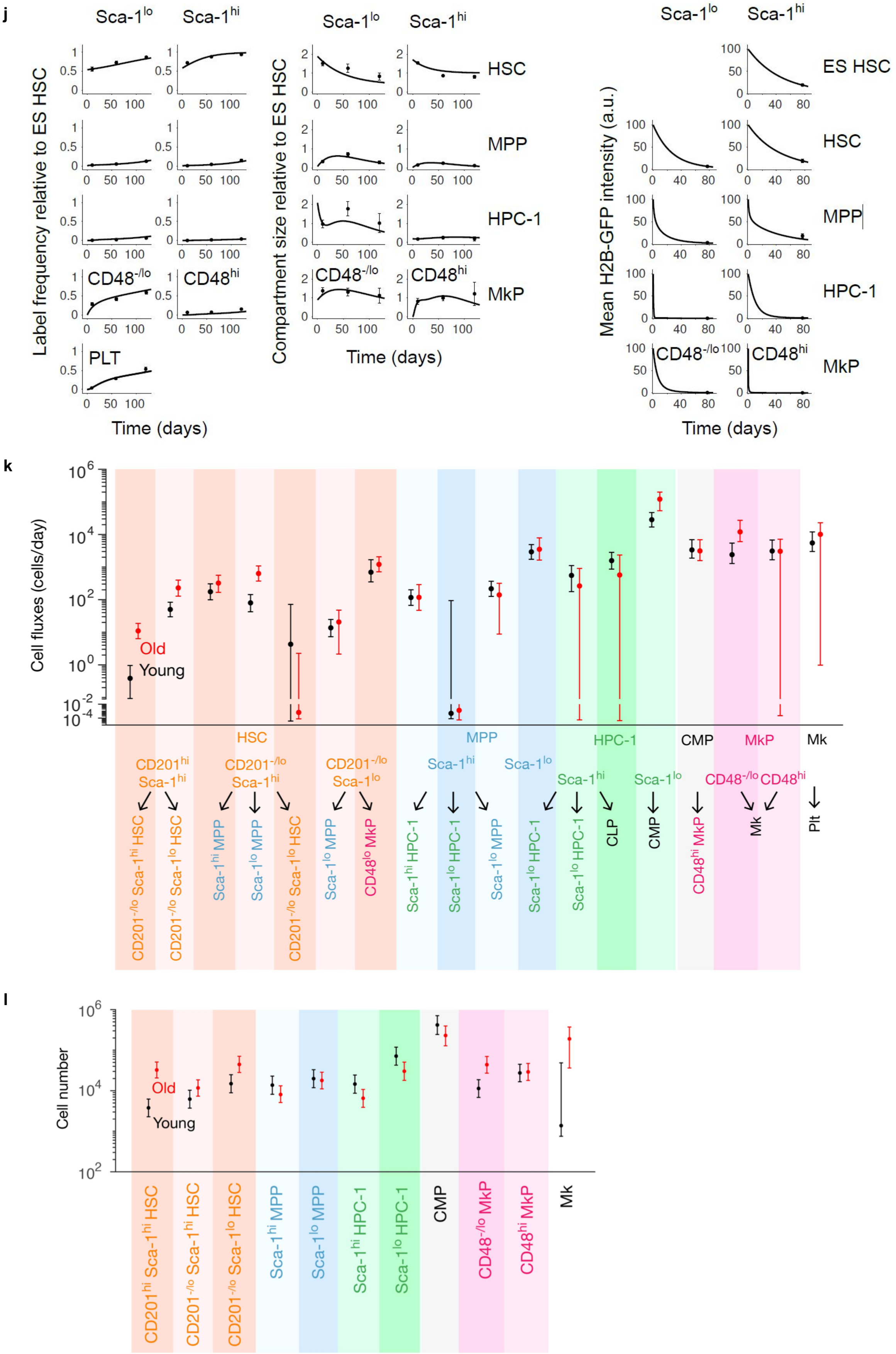

Supplementary Figure 7. Fate mapping in aged and young animals reveals changed dynamics upon ageing. (continued)

j, Model fitting of RFP label propagation (left), compartment size (middle) and H2B-GFP dilution (right) dynamics in aged mice (n = 30 for the left and middle panels and n=3 for the right panel; mean and SEM are shown).

k, Inferred cell fluxes in young (black) and old (red) mice for all differentiation steps.

l, Inferred cell numbers in HSPCs in young (black) and old (red) mice.

In **k** and **l**, dots are for best-fit parameters and error bars indicate 95% prediction bands.

Supplementary Figure 8

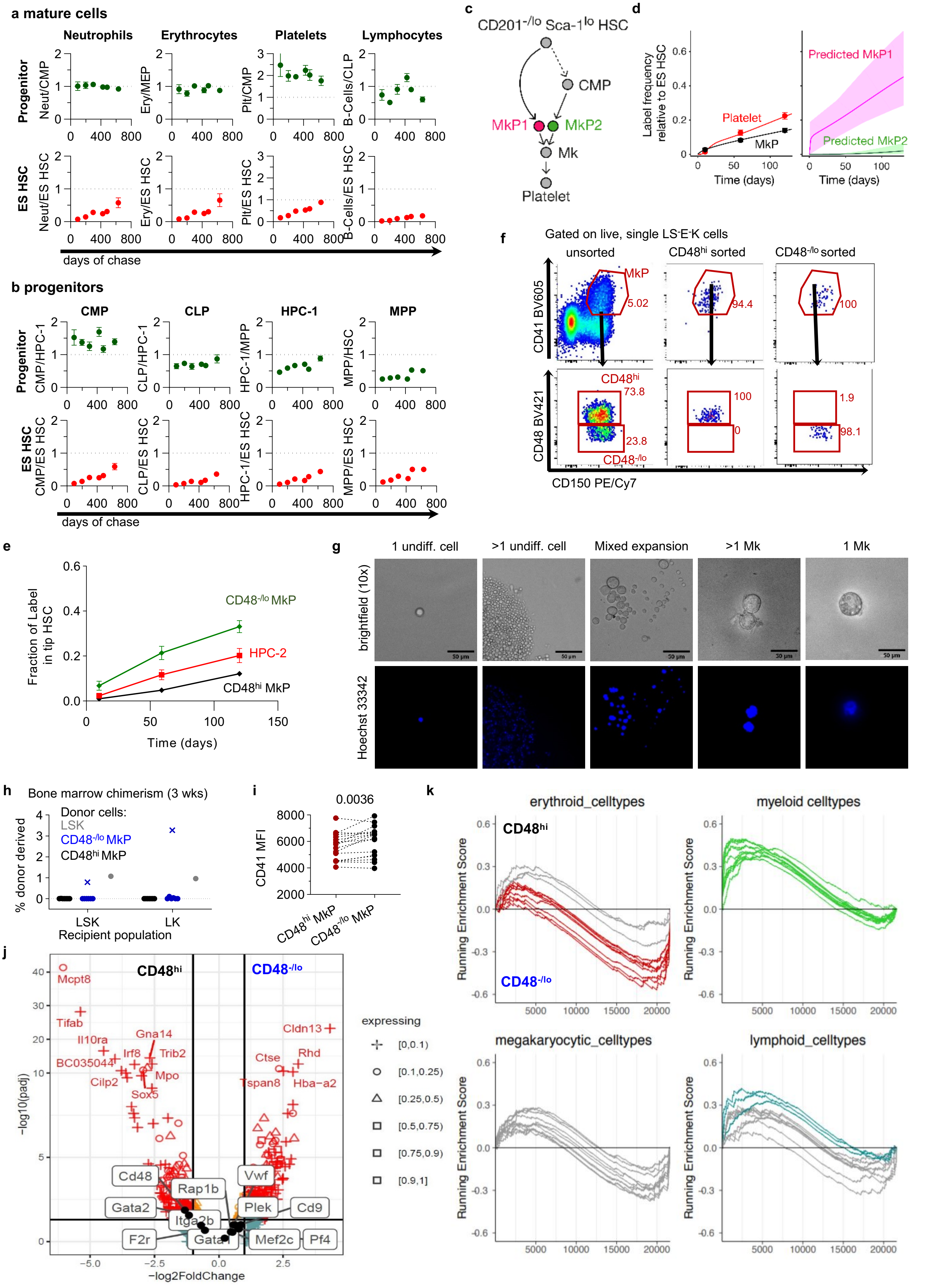

Supplementary Figure 8. Fate mapping reveals two developmentally distinct MkP subpopulations with similar functional potential.

a-b, RFP labeling ratios of mature cells from peripheral blood (a) or BM progenitors (b) to their putative progenitors in the bone marrow (upper row) or ES HSC (lower Platelets are labeled about twice as frequently as CMPs, indicating that platelets may develop independently of CMPs. The ratios of RFP labeling were calculated from *Fgd5*$^{ZsGreen:CreERT2}$/*R26*$^{LSL\text{-}tdRFP}$ mice (n=82 mice, mean ± SEM, same animals as in Figure 1c-e).

c, Hypothetical two-pathway model for MkPs (top), with assumed subpopulations MkP1 (pink) and MkP2 (green).

d, The two-pathway model fits experimental fate mapping data of platelets and MkPs (left), and predicts distinct labeling frequencies of both MkP subpopulations (means and 95% confidence bounds; right).

e, RFP labelling of restricted hematopoietic progenitor 2 (HPC-2, LSK CD48^{hi} CD150^{+}) cells from *Fgd5*$^{ZsGreen:CreERT2}$/*R26*$^{LSL\text{-}tdRFP}$ mice (same as in Figures 3c, 4a and 4b, n=5 animals/ timepoint) compared to RFP label in MkP subpopulations (mean and SEM are shown).

f, Purification strategy and re-analysis of sorted CD48^{hi} and CD48$^{-/lo}$ donor MkPs (LE^{-}S^{-}K: lin^{-} CD201^{-}Sca-1^{-}CD117^{+} BM cells). Frequencies among respective parent populations are shown.

g, Representative examples (upper row) of single MkPs cultured for 5 days and inspected by light microscopy for cell numbers and morphology. Nuclei were stained by adding Hoechst 33342 (5 µg/ml) and evaluated by fluorescence microscopy (lower row). Growth behavior was assigned to 5 categories: 1 undifferentiated cell (1 uc): seeded cell did neither proliferate nor increase in size; >1 uc: seeded cell divided at least once, but daughter cells did not enlarge; mixed expansion: seeded cell divided at least once and daughter cells were heterogeneous in size; >1Mk: seeded cell divided at least once and all daughter cells enlarged and displayed megakaryocytic morphology; 1 Mk: seeded cells increased in size without dividing. Images taken from one experimental replicate (72 MkPs seeded).

Supplementary Figure 8. Fate mapping reveals two developmentally distinct MkP subpopulations with similar functional potential (continued).

h, 2,500 CD48^{hi} or CD48$^{-/lo}$ MkPs were purified from B6.RFP donor animals and transplanted into sublethally-irradiated B6 WT recipient mice (n=7 mice/condition) and contribution to bone marrow HSCs and progenitors was analysed 3 weeks later (individual mice are shown, a single recipient which showed reconstitution of BM LSK cells (x) was excluded from the PB analysis). 2500 transplanted LSK cells served as a positive control (n=1).

i, CD41 expression levels of CD48^{lo} (black) and CD48^{hi} MkPs (red) isolated from *Fgd5*$^{ZsGreen:CreERT2}$/*R26*$^{LSL\text{-}tdRFP}$ mice (same as in Figures 4a and 4b) after 11 days of chase (two-tailed paired Student´s t test; p value shown in graph).

j, k, 4000-20000 CD48^{hi} (n=4 samples) or CD48$^{-/lo}$ (n=3 samples) MkP from WT mice were sorted and subjected to bulk transcriptome analysis. (**j**) Volcano plot shows differentially expressed genes between the two MkP subpopulations. Megakaryocytic signature genes were annotated. The shape of each gene symbol corresponds to its detection rate in the scRNA-seq data of the respective MkP population. (**k**), Gene set enrichment analysis of mature hematopoietic cell lineage signatures extracted from MSigDB. Coloured lines indicate significantly enriched gene sets (adjusted p-value < 0.05). Statistical testing was performed using DESeq2 (j) or GSEA (k) and p-values were adjusted using the Benjamini and Hochberg method.

Supplementary Figure 9

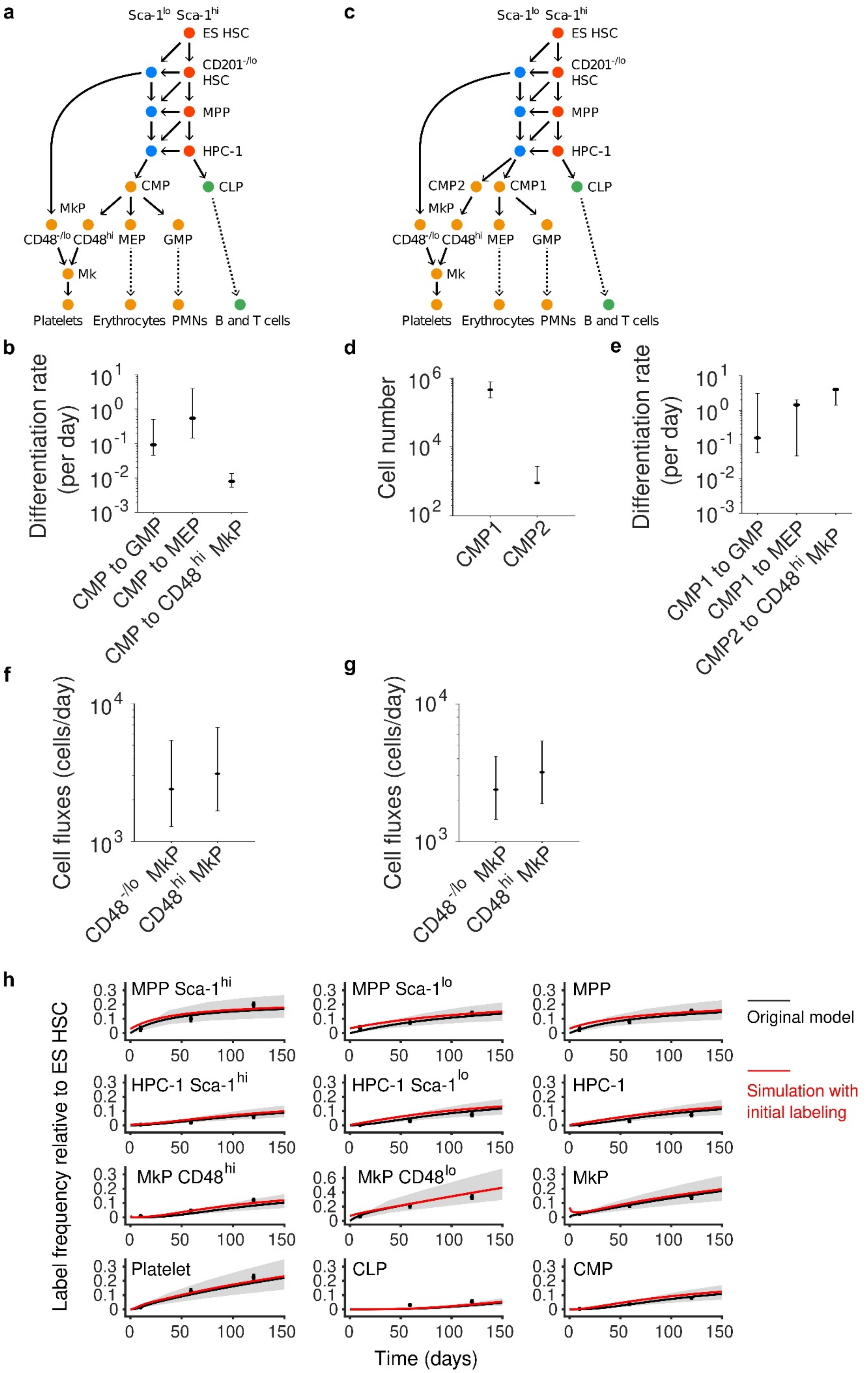

Supplementary Figure 9. Testing alternative model assumptions.

a – g, Modelling CMP heterogeneity.

a, Original model with a homogeneous CMP compartment.

b, Inferred rates of CMP differentiation to GMP, MEP and CD48^{hi} MkP.

c, Model scheme with a heterogeneous CMP compartment. Here, CMP contains a subpopulation (CMP1) that specifically gives rise to GMP and MEP, as well as a second subpopulation (CMP2) that gives rise to MkPs.

d, Inferred cell numbers of CMP subpopulations based on the model in **c**.

e, Inferred differentiation rates of CMP1 → GMP, CMP1 → MEP and CMP2 → CD48^{hi} MkP based on the model in **c**.

f, g, Both the homogeneous (**f**) and heterogeneous (**g**) CMP models show balanced output cell fluxes via CD48$^{-/lo}$ MkP and CD48^{hi} MkP.

In **b** and **d - g**, dots are for best-fit parameters and error bars indicate 95% confidence bands.

h, Computational simulation of how low initial labelling in non-stem-cell compartments affects the fate-mapping dynamics. The simulation takes the measured label frequencies of MPP and MkP at the first time point as the initial labelling values (red line), instead of zeros in the original model (black line). The parameters used are the best fitted ones for the original model (Supplementary Table 1). Black dots: mean values of the label frequencies relative to ES HSC; error bars: standard errors.

Supplementary Figure 10

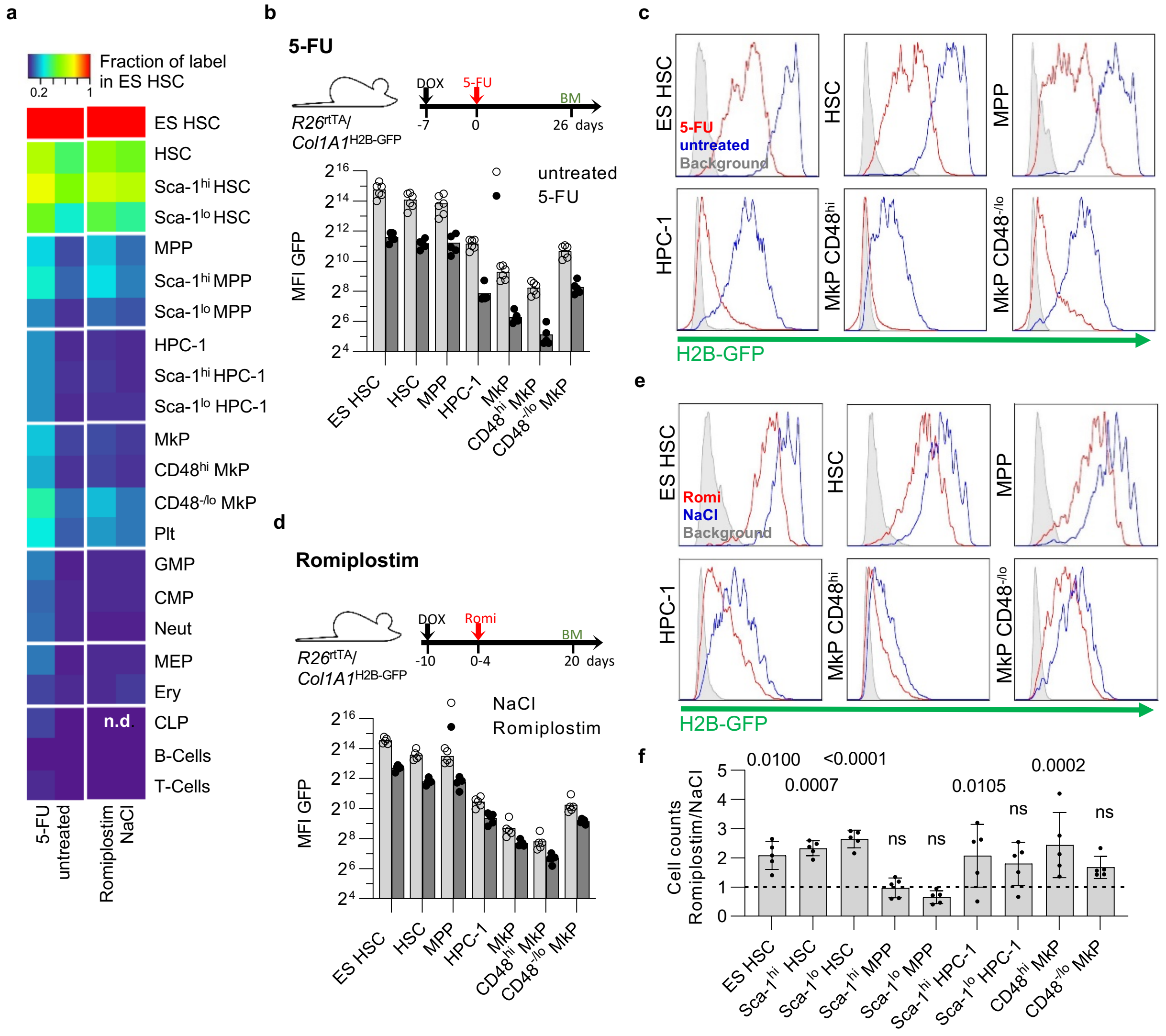

Supplementary Figure 10. Perturbation of hematopoiesis by 5-FU and romiplostim.

a, *Fgd5*$^{\mathrm{ZsGreen:CreERT2}}$/*R26*$^{\mathrm{LSL\text{-}tdRFP}}$ mice were TAM-induced and treated with 5-FU (left heat map, same animals as in Figure 5a) or romiplostim (right heat map, same animals as in Figure 5d). Heat map shows RFP labelling as mean fraction of label in ES HSC.

b and d, *R26*^{rtTA}/*Col1A1*$^{\mathrm{H2B\text{-}GFP}}$ animals were Dox-induced and treated either with 5-FU (b, n=6 mice/condition) or romiplostim (d n=5 mice/condition). BM HSPCs were analysed for H2B-GFP retention at indicated time points. H2B-GFP mean fluorescence intensities (MFI) from individual mice and means thereof (bars) are shown in $\log_2$ display. Population-specific background H2B-GFP fluorescence was subtracted.

c and e, Representative histograms of H2B-GFP fluorescence intensities in selected bone marrow populations of 5-FU (c) or romiplostim (e) treated animals (red histograms) compared to untretaed animals (blue histograms). Background fluorescence in each population is given (grey histograms).

f, Changes in bone marrow population size of *Fgd5*$^{\mathrm{ZsGreen:CreERT2}}$/*R26*$^{\mathrm{LSL\text{-}tdRFP}}$ mice (same as in a) after romiplostim treatment (n=4) compared to saline (NaCl) treated (n=4) control animals (mean ± SEM, one-way ANOVA with Sidak correction, same animals as in Figure 5d; ns, not significant; p values shown in graph). Absolute numbers of lin^- HSPCs isolated from 2 femora, 2 tibiae and 2 pelvises of each individual were normalized to its body weight.

Supplementary Figure 11

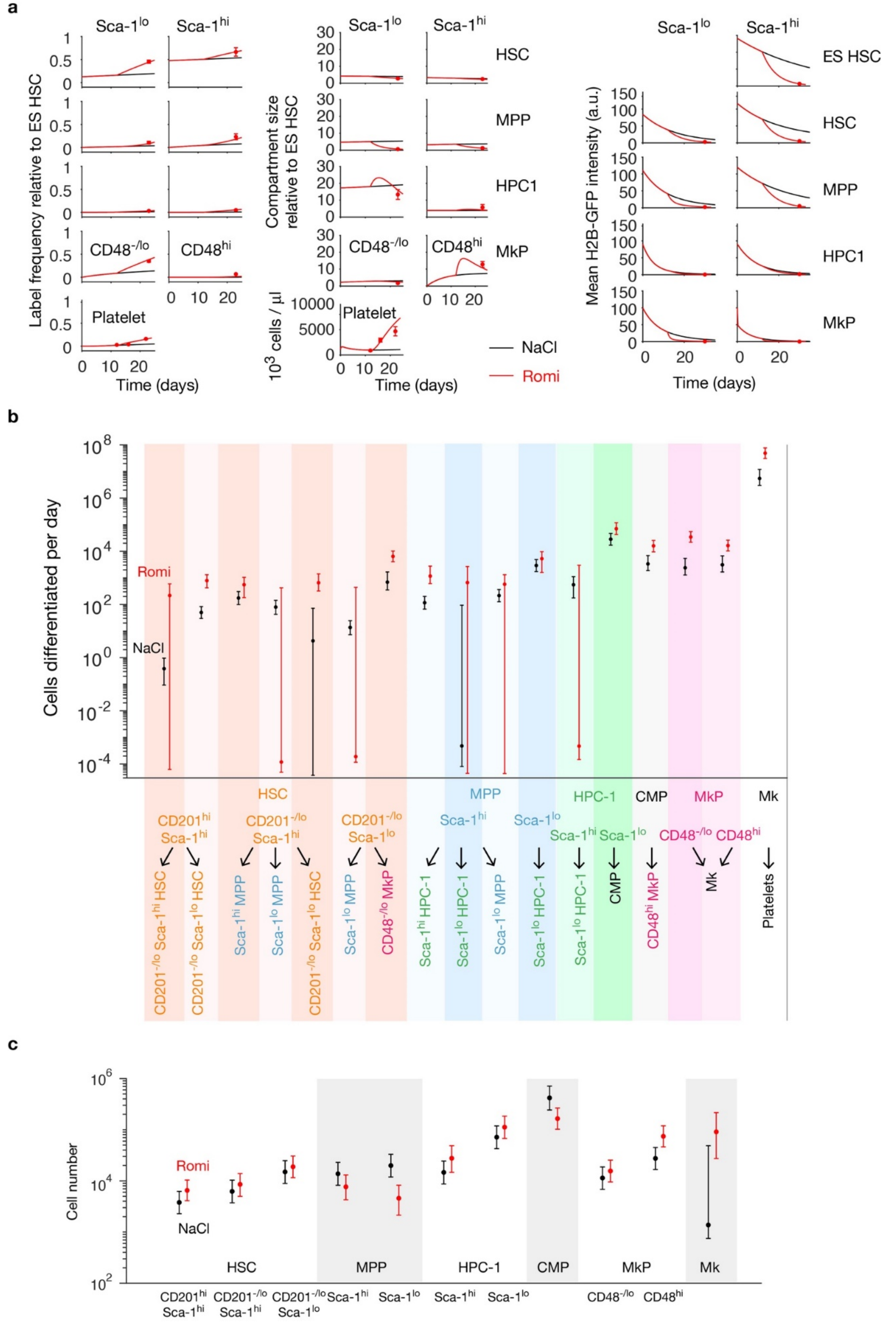

Supplementary Figure 11. Modelling of romiplostim-enhanced thrombopoiesis.

a, Model fitting of RFP label propagation (left), compartment size (middle) and H2B-GFP dilution (right) dynamics in unperturbed (NaCl, black) or romiplostim-treated (red) conditions (n = 4 mice; mean and SEM are shown).

b, Inferred cell fluxes for all differentiation steps.

c, Inferred cell counts of all compartments.

In **b** and **c**, dots are for best-fit parameters and error bars indicate 95% prediction bands.

Supplementary Figure 12

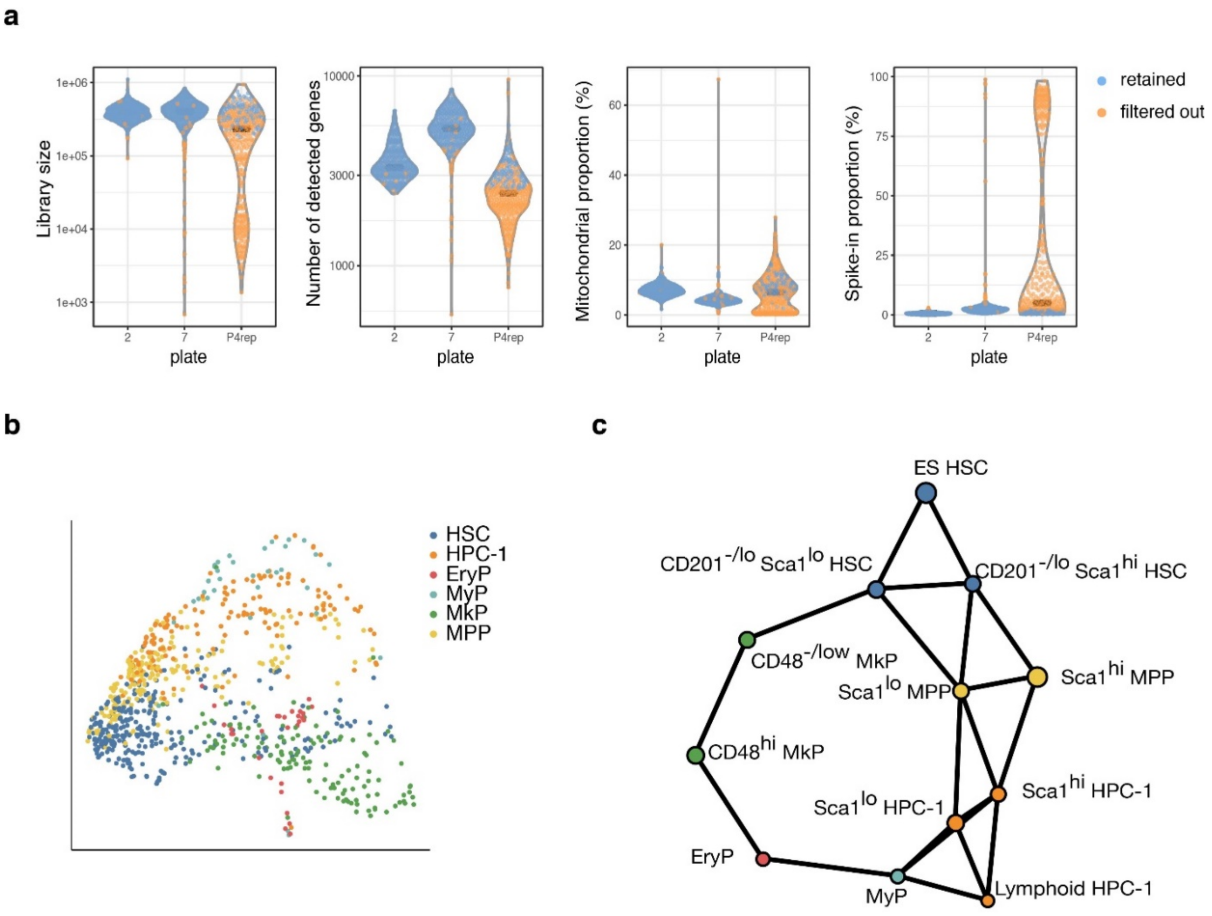

Supplementary Figure 12. Quality control and robustness of scRNA-seq analysis.

a, The quality control metrics of the scRNA-seq analysis, i.e., library size, detected gene counts, mitochondrial reads and spike-in content, are quantified in each sample.

b and c, Mutual nearest neighbor (MNN) correction is used as an alternative method to address the batch effect. Accordingly, the Phate (**a**) and PAGA (**b**) plots were obtained based on MNN.

Supplementary information
Morcos, Li, Munz et al., Fate mapping of hematopoietic stem cells reveals two pathways of native thrombopoiesis

Supplementary methods

Contents

1 Mathematical modeling and inference of lineage pathways 1
- 1.1 Dynamics of hematopoietic cell populations . . . 1
- 1.2 Propagation of fate-mapping label . . . 2
- 1.3 Analysis of heritable label propagation between HSC subpopulations . . . 3
- 1.4 Modeling H2B-GFP dynamics . . . 4
- 1.5 Demonstrating inference of lineage pathways . . . 6

2 Inferring lineage pathways of native hematopoiesis from experimental data 8
- 2.1 Modeling lineage pathways of hematopoietic stem and progenitor cells . . . 8
- 2.2 Quantifying the probability of lineage pathways . . . 10
- 2.3 Model inference including mature cell populations . . . 11
- 2.4 Rate changes upon romiplostim treatment . . . 12

1 Mathematical modeling and inference of lineage pathways

1.1 Dynamics of hematopoietic cell populations

The number of cells in a given hematopoietic population are determined by the balance of cell gain and loss. Cells are gained by influx through differentiation of progenitors and proliferation of cells in the given population; loss occurs by onward differentiation and death. Specifically, all stem and progenitor cells proliferate, whereas the mature cells studied here (e.g.,platelets and erythrocytes) do not proliferate. With the exception of tip stem cells, ES HSCs, all cell populations receive influx. All cell types, including HSCs[1], may die.

The mathematical balance equations for the cell numbers are based on the principle that the flux of cells—the number of cells undergoing a certain process (e.g., cell division) per unit time—is proportional to the number of cells that can potentially undergo this process. The proportionality factor is a rate constant that is the inverse of the average waiting time before a cell commits to the process in question (e.g., the average cell cycle time in the case of cell division). Hence, the balance equations are exact in principle. Their precision in describing the actual biology depends on the appropriate definition of cell populations

and of the waiting time distributions for the processes described. Here, we use the customary exponential waiting time distributions and base the model on a refined definition of the most upstream hematopoietic populations, HSCs, MPPs and HPC-1, that includes, in addition to the usual markers (LSK, CD48 and CD150) also CD201 (EPCR) for HSCs and Sca-1 surface level for HSCs, MPPs and HPC-1. Thus we go well beyond the resolution of previous studies[2,3].

Specifically, HSCs and progenitors can undergo self-renewal (with rate σ), divide asymmetrically (with rate γ), divide into two more differentiated daughter cells (with rate ρ, symmetric differentiating division), differentiate directly (with rate μ) or die (with rate δ) (Supplementary Fig. 4g). Based on these fundamental processes, the population dynamics for a lineage pathway where each product has exactly one precursor are described by the following system of ordinary differential equations:

$$\begin{aligned} \dot{n}_1 &= -(\rho_1 + \mu_1 + \delta_1 - \sigma_1) n_1 \\ \dot{n}_i &= (2\rho_{i-1} + \gamma_{i-1} + \mu_{i-1}) n_{i-1} - (\rho_i + \mu_i + \delta_i - \sigma_i) n_i, \ i \geq 2 \end{aligned} \tag{1}$$

where population 1 are the tip stem cells (ES HSCs) and populations $2, 3, \ldots$ are successive downstream populations. Generalization to pathways where two or more precursors feed into the same product is straightforward. These equations can be rewritten in a simpler form:

$$\begin{aligned} \dot{n}_1 &= -\kappa_1 n_1 \\ \dot{n}_i &= \alpha_{i-1} n_{i-1} - \kappa_i n_i, \ i \geq 2 \end{aligned} \tag{2}$$

using two aggregate parameters:

$$\begin{aligned} \alpha_i &= 2\rho_i + \gamma_i + \mu_i \\ \kappa_i &= \rho_i + \mu_i + \delta_i - \sigma_i \end{aligned} \tag{3}$$

where α_i and κ_i are the total differentiation rate and net cell loss rate of compartment i, respectively. Based on this generic model, in the following sections we develop a quantitative framework of inferring differentiation pathways from the combination of HSC fate-mapping and cell proliferation data.

1.2 Propagation of fate-mapping label

Let m_i and n_i be the labeled and total cell number of population i. The labeling frequency is defined by $f_i \equiv \frac{m_i}{n_i}$. The dynamics of the labeling frequency is governed by,

$$\begin{aligned} \dot{f}_i &= \dot{\left(\frac{m_i}{n_i}\right)} \\ &= \frac{1}{n_i}\dot{m}_i - \frac{m_i}{n_i^2}\dot{n}_i \end{aligned} \tag{4}$$

It is reasonable to assume that heritable labeling (e.g., via RFP expression from the *Rosa26* locus) does not alter cell proliferation, differentiation and death rates. Hence we combine Equation 4 with Equation 2

and obtain

$$\begin{aligned}
\dot{f}_i &= \frac{\alpha_{i-1}}{r_i}(f_{i-1} - f_i),\ i \geq 2 \\
\dot{r}_2 &= \alpha_1 - (\kappa_2 - \kappa_1)\, r_2 \\
\dot{r}_i &= \alpha_{i-1} - \left(\kappa_i - \kappa_{i-1} + \frac{\alpha_{i-2}}{r_{i-1}}\right) r_i,\ i \geq 3
\end{aligned} \tag{5}$$

where for the tip stem cells $f_1 =$ constant, as shown experimentally (Fig. 1d). The new variable r_i denotes the size ratio between neighboring compartments: $r_i \equiv \frac{n_i}{n_{i-1}}$[4]. The generic model can be readily extended to describe more complex lineage trees, e.g. with diverging and converging branches.

1.3 Analysis of heritable label propagation between HSC subpopulations

Functionally different subsets may exist in a phenotypically defined cell population such as HSC. Transitions among these subpopulations may be irreversible or reversible. We now show that reversibility would shape the dynamics of fate-mapping label in a manner that would be easily recognizable in experimental data. To characterize the kinetic behavior of the label propagation in this situation, we consider the simplest case in which two subpopulations A and B give rise to to each other, with rates α_{AB} (for A $\rightarrow$ B) and α_{BA} (for B $\rightarrow$ A). The cell number kinetics of A and B are governed by

$$\begin{aligned}
\dot{n}_{\mathrm{A}} &= \alpha_{\mathrm{BA}} n_{\mathrm{B}} - \kappa_{\mathrm{A}} n_{\mathrm{A}} \\
\dot{n}_{\mathrm{B}} &= \alpha_{\mathrm{AB}} n_{\mathrm{A}} - \kappa_{\mathrm{B}} n_{\mathrm{B}}
\end{aligned} \tag{6}$$

where κ_{A} and κ_{B} are the net cell loss rates of A and B, respectively. Based on Equation 4, the label propagation equations can be derived,

$$\begin{aligned}
\dot{f}_{\mathrm{A}} &= \alpha_{\mathrm{BA}} r_{\mathrm{BA}} \left(f_{\mathrm{B}} - f_{\mathrm{A}}\right) \\
\dot{f}_{\mathrm{B}} &= \frac{\alpha_{\mathrm{AB}}}{r_{\mathrm{BA}}} \left(f_{\mathrm{A}} - f_{\mathrm{B}}\right)
\end{aligned} \tag{7}$$

where $r_{\mathrm{BA}} \equiv \frac{n_{\mathrm{B}}}{n_{\mathrm{A}}}$.

To obtain a steady state for Eqs. 6, the cell loss rates of both subpopulations must be greater than zero, i.e., $\kappa_{\mathrm{A}} > 0$ and $\kappa_{\mathrm{B}} > 0$. Combining Equation 6 and 7 under steady-state condition, the equation for label propagation becomes,

$$\begin{aligned}
\dot{f}_{\mathrm{A}} &= \kappa_{\mathrm{A}} \left(f_{\mathrm{B}} - f_{\mathrm{A}}\right) \\
\dot{f}_{\mathrm{B}} &= \kappa_{\mathrm{B}} \left(f_{\mathrm{A}} - f_{\mathrm{B}}\right)
\end{aligned} \tag{8}$$

The analytical solution of this equation is,

$$\begin{aligned}
f_{\mathrm{A}}(t) &= \frac{f_{\mathrm{B0}}\kappa_{\mathrm{A}} + f_{\mathrm{A0}}\kappa_{\mathrm{B}}}{\kappa_{\mathrm{A}} + \kappa_{\mathrm{B}}} + \frac{\kappa_{\mathrm{A}}}{\kappa_{\mathrm{A}} + \kappa_{\mathrm{B}}}(f_{\mathrm{A0}} - f_{\mathrm{B0}})e^{-(\kappa_{\mathrm{A}}+\kappa_{\mathrm{B}})t} \\
f_{\mathrm{B}}(t) &= \frac{f_{\mathrm{B0}}\kappa_{\mathrm{A}} + f_{\mathrm{A0}}\kappa_{\mathrm{B}}}{\kappa_{\mathrm{A}} + \kappa_{\mathrm{B}}} - \frac{\kappa_{\mathrm{B}}}{\kappa_{\mathrm{A}} + \kappa_{\mathrm{B}}}(f_{\mathrm{A0}} - f_{\mathrm{B0}})e^{-(\kappa_{\mathrm{A}}+\kappa_{\mathrm{B}})t}
\end{aligned} \tag{9}$$

where f_{A0} and f_{B0} are the initial (at t = 0) labeling frequencies of A and B, respectively.

The solution shows that the label frequencies of subpopulations A and B will eventually equilibrate at an intermediate value between their initial label frequencies (the first term in Equation 9), which is the average of their initial labeling weighted by the net cell loss rates (κ_A and κ_B). As a consequence, the two subpopulations reach the labeling equilibrium in opposite manners. Specifically, if A is preferentially labeled initially, i.e., $f_{A0} > f_{B0}$, then the label frequency of B will increase with time, whereas that of A will decline. By contrast, irreversible transition, e.g., A $\rightarrow$ B, implies that A will maintain a constant labeling (its initial value) over time, and B will increase its label frequency and finally equilibrate with that of A. This feature is governed by the following equation (setting κ_A to be zero in Equation 9),

$$\begin{aligned} f_A(t) &= f_{A0} \\ f_B(t) &= f_{A0} - (f_{A0} - f_{B0})e^{-\kappa_B t} \end{aligned} \tag{10}$$

Taken together, reversible and irreversible transition schemes among cell subpopulations can be well distinguished by the directions of label frequencies approaching equilibrium. If subpopulations are initially labeled differently, then a reversible transition between them causes a decrease of labeling frequency in the initially more strongly labeled one. Absence of this feature, as in our data on HSC subpopulations, rules out reversibility.

1.4 Modeling H2B-GFP dynamics

To infer cell proliferation rates from the H2B-GFP dilution data, we model the temporal changes of the mean H2B-GFP intensity (total H2B-GFP intensity divided by cell number) in each cell population, which can be readily obtained from experimental data, i.e., taking the mean value of the H2B-GFP distribution followed by proper scaling for the convenience of numerical computation (mean value of the raw data divided by 1000 in this work). The cell number dynamics is given by Equation 2, so next we derive the equations for total H2B-GFP intensity in each compartment.

We assume that H2B-GFP turnover and basal production change the fluorescence intensity of all the cell populations uniformly, whereas cell proliferation, differentiation and death do so in a population-specific manner. Let $X_i(t)$ be the total H2B-GFP intensity of compartment i at time t. During time increment Δt, H2B-GFP turnover and basal production cause the change in total intensity by,

$$\Delta X_{i,\text{prod-deg}} = n_i(t) v \Delta t - k_d \Delta t X_i \tag{11}$$

where $n_i(t)$ is the cell number of compartment i, v the basal H2B-GFP production rate per cell and k_d the turnover rate.

Symmetric self-renewal redistributes existing H2B-GFP into more cells in the same population, thus altering the relative (i.e., per cell) but not the total H2B-GFP intensity of the population. By contrast, cell death or differentiation into the next population, i.e., $i \rightarrow i + 1$, cause H2B-GFP loss from population i. The intensity loss equals the number of cells that leave the compartment times the H2B-GFP intensity taken away per cell. For example, the cell number loss after Δt is $\gamma_i n_i(t) \Delta t$ due to asymmetric differentiation. The H2B-GFP level loss per cell is half of the current level, i.e., $\frac{1}{2}\frac{X_i(t)}{n_i(t)}$ because only one of the two daughter cells goes to the next population. The change in total H2B-GFP level is then $-\gamma_i n_i(t) \Delta t \frac{1}{2}\frac{X_i(t)}{n_i(t)}$. For cells undergoing death, symmetric or direct differentiation, all H2B-GFP of these cells is lost from population i.

Thus, the total H2B-GFP loss after Δt for compartment i is,

$$\Delta X_{i\to i+1} = -\gamma_i n_i(t)\Delta t \frac{1}{2}\frac{X_i(t)}{n_i(t)} - (\rho_i + \mu_i + \delta_i)\, n_i(t)\Delta t \frac{X_i(t)}{n_i(t)} \tag{12}$$

Furthermore, each population, except for the tip stem cells, receives H2B-GFP from its direct upstream population via differentiation. In a similar way, we formulate this effect as,

$$\begin{aligned}\Delta X_{i-1\to i} &= \gamma_{i-1} n_{i-1}(t)\Delta t \frac{1}{2}\frac{X_{i-1}(t)}{n_{i-1}(t)} \\ &+ 2\rho_{i-1} n_{i-1}(t)\Delta t \frac{1}{2}\frac{X_{i-1}(t)}{n_{i-1}(t)} \\ &+ \mu_{i-1} n_{i-1}(t)\Delta t \frac{X_{i-1}(t)}{n_{i-1}(t)}\end{aligned} \tag{13}$$

Therefore, the total H2B-GFP level of compartment i at time $t + \Delta t$ is,

$$\begin{aligned}X_i(t+\Delta t) &= X_i(t) + \Delta X_{i,\mathrm{prod-deg}} + \Delta X_{i\to i+1} + \Delta X_{i-1\to i} \\ &= X_i(t) + n_i v \Delta t - k_\mathrm{d}\Delta t X_i(t) \\ &- \gamma_i n_i(t)\Delta t \frac{1}{2}\frac{X_i(t)}{n_i(t)} - (\rho_i + \mu_i + \delta_i) n_i(t)\Delta t \frac{X_i(t)}{n_i(t)} \\ &+ \gamma_{i-1} n_{i-1}(t)\Delta t \frac{1}{2}\frac{X_{i-1}(t)}{n_{i-1}(t)} \\ &+ 2\rho_{i-1} n_{i-1}(t)\Delta t \frac{1}{2}\frac{X_{i-1}(t)}{n_{i-1}(t)} \\ &+ \mu_{i-1} n_{i-1}(t)\Delta t \frac{X_{i-1}(t)}{n_{i-1}(t)}\end{aligned} \tag{14}$$

The dynamics of the mean H2B-GFP intensity $x_i(t) \equiv \frac{X_i(t)}{n_i(t)}$ obeys,

$$\begin{aligned}\dot{x}_i &= \left(\frac{\dot{X_i}}{n_i}\right) \\ &= \frac{1}{n_i}\dot{X}_i - \frac{X_i}{n_i^2}\dot{n}_i\end{aligned} \tag{15}$$

Combining this with Equation 1, we obtain

$$\dot{x}_i = v - k_\mathrm{d} x_i - \frac{2\sigma_i + \gamma_i}{2} x_i + \frac{2\rho_{i-1} + \gamma_{i-1}}{r_i}\left(\frac{x_{i-1}}{2} - x_i\right) + \frac{\mu_{i-1}}{r_i}(x_{i-1} - x_i) \tag{16}$$

This equation shows how each elementary process contributes to the H2B-GFP dynamics. First, cell death, symmetric and direct differentiation of a given population i do not affect its own mean H2B-GFP level (none of the parameters δ_i, ρ_i or μ_i appear in the equation). Second, H2B-GFP intensity is diluted by symmetric self-renewal and asymmetric differentiation. Third, cells entering via differentiation from the upstream population can either increase or reduce the mean H2B-GFP level, depending on the intensity difference. Finally, H2B-GFP dilution is also determined by its own degradation (with k_d). For all parameter inferences in this paper, we fixed k_d to its previously determined value in murine HSCs, corresponding to a half-life of 42 days[5], thus improving inference of the relevant rate constants of HSC and progenitor

proliferation, differentiation and death.

We rewrite Equation 11 using the aggregate parameters defined in Equation 3

$$\begin{aligned} \dot{x}_1 &= v - k_\mathrm{d} x_1 - \frac{\lambda_1}{2} x_1 \\ \dot{x}_i &= v - k_\mathrm{d} x_i + \frac{\alpha_{i-1} + \mu_{i-1}}{2 r_i} x_{i-1} - \left(\frac{\lambda_i}{2} + \frac{\alpha_{i-1}}{r_i} \right) x_i, \; i \geq 2 \end{aligned} \tag{17}$$

and introduce another aggregate parameter λ_i, the total self-renewal rate

$$\lambda_i = 2\sigma_i + \gamma_i \tag{18}$$

In summary, we obtain a system of differential equations for cell numbers (Equation 2), frequencies of fate-mapping label (Equation 5) and average H2B-GFP intensities (Equation 12) in hematopoietic cell populations. The key parameters characterizing the dynamics of cell proliferation and differentiation—total differentiation rates α_i, direct differentiation rates μ_i, net loss rates κ_i and total self-renewal rates λ_i—can all be time-dependent (e.g., change with increasing age of the animal).

1.5 Demonstrating inference of lineage pathways

Label propagation and H2B-GFP dilution experiments yield, respectively, differentiation and proliferation rates of stem and progenitor cells. We therefore reasoned that the combination of the two types of experimental data will allow inference of both differentiation and proliferation rates in a complex lineage tree. We have advanced this argument recently for the combination of HSC fate mapping and BrdU incorporation (instead of H2B-GFP dilution)[4]. The principal new aspect in this paper is that we show that combining these types of experimental data also yields information on the topology of the underlying lineage tree. To show this, we first developed the idea with a toy model (Supplementary Fig. 4). To this end, we simulated data for a known lineage topology, and then asked whether model selection based on these *in silico* data with a broader set of possible lineage topologies indeed identifies the correct topology (ground truth).

We collect the equations from Equation 5 and 17,

$$\begin{aligned} \dot{f}_i &= \frac{\alpha_{i-1}}{r_i} (f_{i-1} - f_i), \; i \geq 2 \\ \dot{r}_2 &= \alpha_1 - (\kappa_2 - \kappa_1)\, r_2 \\ \dot{r}_i &= \alpha_{i-1} - \left(\kappa_i - \kappa_{i-1} + \frac{\alpha_{i-2}}{r_{i-1}} \right) r_i, \; i \geq 3 \\ \dot{x}_1 &= v - k_\mathrm{d} x_1 - \frac{\lambda_1}{2} x_1 \\ \dot{x}_i &= v - k_\mathrm{d} x_i + \frac{\alpha_{i-1} + \mu_{i-1}}{2 r_i} x_{i-1} - \left(\frac{\lambda_i}{2} + \frac{\alpha_{i-1}}{r_i} \right) x_i, \; i \geq 2 \end{aligned} \tag{19}$$

where

$$\begin{aligned} \alpha_i &= 2\rho_i + \gamma_i + \mu_i \\ \kappa_i &= \rho_i + \mu_i + \delta_i - \sigma_i \\ \lambda_i &= 2\sigma_i + \gamma_i \end{aligned} \tag{20}$$

The value of total net cell loss κ_i can be positive or negative. To be more practical, we substitute κ_i with

other naturally positive parameters,

$$\kappa_i = \frac{1}{2}(\alpha_i + \mu_i - \lambda_i) + \delta_i \tag{21}$$

According to Equation 19, each compartment is governed by 4 parameters (α_i, λ_i, μ_i and δ_i), while there are 5 underlying elementary parameters (ρ_i, γ_i, σ_i, μ_i and δ_i). Thus, model inference has a natural limitation in identifying each individual fundamental parameters. However, inference based on the aggregate parameters is sufficient for identifying differentiation pathways.

Next, we test this framework by inferring differentiation pathways based on *in silico* data. We consider a lineage tree with one stem cell (S), two progenitor cells (P1 and P2) and three mature cells (M1, M2 and M3) with a unique pathway topology (Supplementary Fig. 4b) and assign physiologically reasonable parameters. We obtained the *in silico* data by adding white noise to the simulated data (label propagation, compartment size and H2B-GFP dilution data), with different coefficients of variance for different time points. We enumerated 6 possible model schemes (without lineage convergence), and performed statistical model selection based on the simulated data (Supplementary Fig. 4c). The equations for the true model scheme are:

$$\begin{aligned}
\dot{f}_{\mathrm{p1}} &= \frac{\alpha_{\mathrm{s}\to\mathrm{p1}}}{r_{\mathrm{p1}}}(f_{\mathrm{s}} - f_{\mathrm{p1}}) \\
\dot{f}_{\mathrm{p2}} &= \frac{\alpha_{\mathrm{s}\to\mathrm{p2}}}{r_{\mathrm{p2}}}(f_{\mathrm{s}} - f_{\mathrm{p2}}) \\
\dot{f}_{\mathrm{m1}} &= \frac{\alpha_{\mathrm{p1}\to\mathrm{m1}}}{r_{\mathrm{m1}}}(f_{\mathrm{p1}} - f_{\mathrm{m1}}) \\
\dot{f}_{\mathrm{m2}} &= \frac{\alpha_{\mathrm{p1}\to\mathrm{m2}}}{r_{\mathrm{m2}}}(f_{\mathrm{p1}} - f_{\mathrm{m2}}) \\
\dot{f}_{\mathrm{m3}} &= \frac{\alpha_{\mathrm{p2}\to\mathrm{m3}}}{r_{\mathrm{m3}}}(f_{\mathrm{p2}} - f_{\mathrm{m3}}) \\
\dot{r}_{\mathrm{p1}} &= \alpha_{\mathrm{s}\to\mathrm{p1}} - (\kappa_{\mathrm{p1}} - \kappa_{\mathrm{s}})r_{\mathrm{p1}} \\
\dot{r}_{\mathrm{p2}} &= \alpha_{\mathrm{s}\to\mathrm{p2}} - (\kappa_{\mathrm{p2}} - \kappa_{\mathrm{s}})r_{\mathrm{p2}} \\
\dot{r}_{\mathrm{m1}} &= \alpha_{\mathrm{p1}\to\mathrm{m1}} - (\kappa_{\mathrm{m1}} - \kappa_{\mathrm{p1}} + \frac{\alpha_{\mathrm{s}\to\mathrm{p1}}}{r_{\mathrm{p1}}})r_{\mathrm{m1}} \\
\dot{r}_{\mathrm{m2}} &= \alpha_{\mathrm{p1}\to\mathrm{m2}} - (\kappa_{\mathrm{m2}} - \kappa_{\mathrm{p1}} + \frac{\alpha_{\mathrm{s}\to\mathrm{p1}}}{r_{\mathrm{p1}}})r_{\mathrm{m2}} \\
\dot{r}_{\mathrm{m3}} &= \alpha_{\mathrm{p2}\to\mathrm{m3}} - (\kappa_{\mathrm{m3}} - \kappa_{\mathrm{p2}} + \frac{\alpha_{\mathrm{s}\to\mathrm{p2}}}{r_{\mathrm{p2}}})r_{\mathrm{m3}} \\
\dot{x}_{\mathrm{s}} &= v - k_{\mathrm{d}}x_{\mathrm{s}} - \frac{\lambda_{\mathrm{s}}}{2}x_{\mathrm{s}} \\
\dot{x}_{\mathrm{p1}} &= v - k_{\mathrm{d}}x_{\mathrm{p1}} + \frac{\alpha_{\mathrm{s}\to\mathrm{p1}} + \mu_{\mathrm{s}\to\mathrm{p1}}}{2r_{\mathrm{p1}}}x_{\mathrm{s}} - \left(\frac{\lambda_{\mathrm{p1}}}{2} + \frac{\alpha_{\mathrm{s}\to\mathrm{p1}}}{r_{\mathrm{p1}}}\right)x_{\mathrm{p1}} \\
\dot{x}_{\mathrm{p2}} &= v - k_{\mathrm{d}}x_{\mathrm{p2}} + \frac{\alpha_{\mathrm{s}\to\mathrm{p2}} + \mu_{\mathrm{s}\to\mathrm{p2}}}{2r_{\mathrm{p2}}}x_{\mathrm{s}} - \left(\frac{\lambda_{\mathrm{p2}}}{2} + \frac{\alpha_{\mathrm{s}\to\mathrm{p2}}}{r_{\mathrm{p2}}}\right)x_{\mathrm{p2}}
\end{aligned} \tag{22}$$

where we substitute the net cell loss rates κ of the stem and progenitor cells using the following equations,

$$\begin{aligned}
\kappa_{\mathrm{s}} &= (\alpha_{\mathrm{s}\to\mathrm{p1}} + \alpha_{\mathrm{s}\to\mathrm{p2}} + \mu_{\mathrm{s}\to\mathrm{p1}} + \mu_{\mathrm{s}\to\mathrm{p2}} - \lambda_{\mathrm{s}})/2 + \delta_{\mathrm{s}} \\
\kappa_{\mathrm{p1}} &= (\alpha_{\mathrm{p1}\to\mathrm{m1}} + \alpha_{\mathrm{p1}\to\mathrm{m2}} + \mu_{\mathrm{p1}\to\mathrm{m1}} + \mu_{\mathrm{p1}\to\mathrm{m2}} - \lambda_{\mathrm{p1}})/2 + \delta_{\mathrm{p1}} \\
\kappa_{\mathrm{p2}} &= (\alpha_{\mathrm{p2}\to\mathrm{m3}} + \mu_{\mathrm{p2}\to\mathrm{m3}} - \lambda_{\mathrm{p2}})/2 + \delta_{\mathrm{p2}}
\end{aligned} \tag{23}$$

Here, we do not explicitly consider the proliferation or death of the mature cells but simply quantify their net

loss rate (in the case of non-proliferating mature cells, the death rate will equal the net loss rate).

The model selection successfully discovered the true model (Supplementary Fig. 4d and e). In stark contrast, the true model could not be singled out by inference without the proliferation data (Supplementary Fig. 4f). To conclude, combining fate-mapping and cell proliferation data allows both identification of lineage topology and quantification the cell fluxes in the lineage pathways.

2 Inferring lineage pathways of native hematopoiesis from experimental data

2.1 Modeling lineage pathways of hematopoietic stem and progenitor cells

To address the experimental data on HSC fate mapping, augmented by tracking of mitotic history of HSCs and progenitors via H2B-GFP, we devised a family of models, with each HSC and progenitor population divided into Sca-1 high and Sca-1 low subpopulations (Supplementary Fig. 4h). HSC subpopulations are further divided by CD201 level. The CD201$^{-/lo}$ Sca-1^{lo} subpopulation has very few cells, so it is not considered in the models. To identify the differentiation pathways, we performed statistical model selection against the experimental data. The equations for the full model with all possible differentiation and Sca-1

transition steps read:

$$
\begin{aligned}
\dot{f}_{2h} &= \frac{\alpha_{1hh}}{r_2}(f_{1h} - f_{2h}) + \phi_{2l}s_2(f_{2l} - f_{2h}) \\
\dot{f}_{2l} &= \frac{\alpha_{1hl}}{r_2 s_2}(f_{1h} - f_{2l}) + \frac{\phi_{2h}}{s_2}(f_{2h} - f_{2l}) \\
\dot{f}_{3h} &= \frac{\alpha_{2hh}}{r_3}(f_{2h} - f_{3h}) + \frac{\alpha_{2lh}s_2}{r_3}(f_{2l} - f_{3h}) + \phi_{3l}s_3(f_{3l} - f_{3h}) \\
\dot{f}_{3l} &= \frac{\alpha_{2ll}s_2}{r_3 s_3}(f_{2l} - f_{3l}) + \frac{\alpha_{2hl}}{r_3 s_3}(f_{2h} - f_{3l}) + \frac{\phi_{3h}}{s_3}(f_{3h} - f_{3l}) \\
\dot{f}_{4h} &= \frac{\alpha_{3hh}}{r_4}(f_{3h} - f_{4h}) + \frac{\alpha_{3lh}s_3}{r_4}(f_{3l} - f_{4h}) + \phi_{4l}s_4(f_{4l} - f_{4h}) \\
\dot{f}_{4l} &= \frac{\alpha_{3ll}s_3}{r_4 s_4}(f_{3l} - f_{4l}) + \frac{\alpha_{3hl}}{r_4 s_4}(f_{3h} - f_{4l}) + \frac{\phi_{4h}}{s_4}(f_{4h} - f_{4l}) \\
\dot{f}_{cmp} &= \frac{\alpha_{4h\to cmp}}{r_{cmp}}(f_{4h} - f_{cmp}) + \frac{\alpha_{4l\to cmp}s_4}{r_{cmp}}(f_{4l} - f_{cmp}) \\
\dot{f}_{clp} &= \frac{\alpha_{4h\to clp}}{r_{clp}}(f_{4h} - f_{clp}) + \frac{\alpha_{4l\to clp}s_4}{r_{clp}}(f_{4l} - f_{clp}) \\
\dot{r}_2 &= \alpha_{1hh} - (\kappa_{2h} - \kappa_{1h} - \phi_{2l}s_2)r_2 \\
\dot{r}_3 &= \alpha_{2hh} + \alpha_{2lh}s_2 - (\kappa_{3h} - \kappa_{2h} - \phi_{3l}s_3 + \frac{\alpha_{1hh}}{r_2} + \phi_{2l}s_2)r_3 \\
\dot{r}_4 &= \alpha_{3hh} + \alpha_{3lh}s_3 - (\kappa_{4h} - \kappa_{3h} - \phi_{4l}s_4 + \frac{\alpha_{2hh}}{r_3} + \frac{\alpha_{2lh}s_2}{r_3} + \phi_{3l}s_3)r_4 \\
\dot{s}_2 &= \phi_{2h} - (\kappa_{2l} - \kappa_{2h})s_2 - \phi_{2l}s_2^2 + (\alpha_{1hl} - \alpha_{1hh}s_2)/r_2 \\
\dot{s}_3 &= \phi_{3h} - (\kappa_{3l} - \kappa_{3h})s_3 - \phi_{3l}s_3^2 + (\alpha_{2hl} + \alpha_{2ll}s_2 - \alpha_{2hh}s_3 - \alpha_{2lh}s_2 s_3)/r_3 \\
\dot{s}_4 &= \phi_{4h} - (\kappa_{4l} - \kappa_{4h})s_4 - \phi_{4l}s_4^2 + (\alpha_{3hl} + \alpha_{3ll}s_3 - \alpha_{3hh}s_4 - \alpha_{3lh}s_3 s_4)/r_4 \\
\dot{x}_{1h} &= v - k_d x_{1h} - \frac{\lambda_{1h}}{2}x_{1h} \\
\dot{x}_{2h} &= v - k_d x_{2h} + \frac{\alpha_{1hh} + \mu_{1hh}}{2r_2}x_{1h} + \phi_{2l}s_2 x_{2l} - \left(\frac{\lambda_{2h}}{2} + \phi_{2l}s_2 + \frac{\alpha_{1hh}}{r_2}\right)x_{2h} \\
\dot{x}_{2l} &= v - k_d x_{2l} + \frac{\alpha_{1hl} + \mu_{1hl}}{2r_2 s_2}x_{1h} + \frac{\phi_{2h}}{s_2}x_{2h} - \left(\frac{\lambda_{2l}}{2} + \frac{\phi_{2h}}{s_2} + \frac{\alpha_{1hl}}{r_2 s_2}\right)x_{2l} \\
\dot{x}_{3h} &= v - k_d x_{3h} + \frac{\alpha_{2hh} + \mu_{2hh}}{2r_3}x_{2h} + \frac{(\alpha_{2lh} + \mu_{2lh})s_2}{2r_3}x_{2l} + \phi_{3l}s_3 x_{3l} - \left(\frac{\lambda_{3h}}{2} + \phi_{3l}s_3 + \frac{\alpha_{2hh} + \alpha_{2lh}s_2}{r_3}\right)x_{3h} \\
\dot{x}_{3l} &= v - k_d x_{3l} + \frac{(\alpha_{2ll} + \mu_{2ll})s_2}{2r_3 s_3}x_{2l} + \frac{\alpha_{2hl} + \mu_{2hl}}{2r_3 s_3}x_{2h} + \frac{\phi_{3h}}{s_3}x_{3h} - \left(\frac{\lambda_{3l}}{2} + \frac{\phi_{3h}}{s_3} + \frac{\alpha_{2ll}s_2 + \alpha_{2hl}}{r_3 s_3}\right)x_{3l} \\
\dot{x}_{4h} &= v - k_d x_{4h} + \frac{\alpha_{3hh} + \mu_{3hh}}{2r_4}x_{3h} + \frac{(\alpha_{3lh} + \mu_{3lh})s_3}{2r_4}x_{3l} + \phi_{4l}s_4 x_{4l} - \left(\frac{\lambda_{4h}}{2} + \phi_{4l}s_4 + \frac{\alpha_{3hh} + \alpha_{3lh}s_3}{r_4}\right)x_{4h} \\
\dot{x}_{4l} &= v - k_d x_{4l} + \frac{(\alpha_{3ll} + \mu_{3ll})s_3}{2r_4 s_4}x_{3l} + \frac{\alpha_{3hl} + \mu_{3hl}}{2r_4 s_4}x_{3h} + \frac{\phi_{4h}}{s_4}x_{4h} - \left(\frac{\lambda_{4l}}{2} + \frac{\phi_{4h}}{s_4} + \frac{\alpha_{3ll}s_3 + \alpha_{3hl}}{r_4 s_4}\right)x_{4l}
\end{aligned}
\tag{24}
$$

where

$$
\begin{aligned}
\kappa_{1\text{h}} &= (\alpha_{1hh} + \alpha_{1hl} + \mu_{1hh} + \mu_{1hl} - \lambda_{1h})/2 + \delta_{1h} \\
\kappa_{2\text{h}} &= (\alpha_{2\text{hh}} + \alpha_{2\text{hl}} + \mu_{2\text{hh}} + \mu_{2\text{hl}} - \lambda_{2\text{h}})/2 + \delta_{2\text{h}} + \phi_{2\text{h}} \\
\kappa_{3\text{h}} &= (\alpha_{3\text{hh}} + \alpha_{3\text{hl}} + \mu_{3\text{hh}} + \mu_{3\text{hl}} - \lambda_{3\text{h}})/2 + \delta_{3\text{h}} + \phi_{3\text{h}} \\
\kappa_{4\text{h}} &= (\alpha_{4\text{h}\to\text{cmp}} + \alpha_{4\text{h}\to\text{clp}} + \mu_{4\text{h}\to\text{cmp}} + \mu_{4\text{h}\to\text{clp}} - \lambda_{4\text{h}})/2 + \delta_{4\text{h}} + \phi_{4\text{h}} \\
\kappa_{2\text{l}} &= (\alpha_{2\text{ll}} + \alpha_{2\text{lh}} + \mu_{2\text{ll}} + \mu_{2\text{lh}} - \lambda_{2\text{l}})/2 + \delta_{2\text{l}} + \phi_{2\text{l}} \\
\kappa_{3\text{l}} &= (\alpha_{3\text{ll}} + \alpha_{3\text{lh}} + \mu_{3\text{ll}} + \mu_{3\text{lh}} - \lambda_{3\text{l}})/2 + \delta_{3\text{l}} + \phi_{3\text{l}} \\
\kappa_{4\text{l}} &= (\alpha_{4\text{l}\to\text{cmp}} + \alpha_{4\text{l}\to\text{clp}} + \mu_{4\text{l}\to\text{cmp}} + \mu_{4\text{l}\to\text{clp}} - \lambda_{4\text{l}})/2 + \delta_{4\text{l}} + \phi_{4\text{l}}
\end{aligned}
\tag{25}
$$

The subscript i in the model variables and parameters ranges from 1 to 4, representing cell compartments CD201^{hi} HSC, CD201$^{\text{-/lo}}$ HSC, MPP and HPC-1, respectively. In addition, the subscripts ih and il stand for compartment i Sca-1 high and Sca-1 low, respectively. The variable r_i is the compartment size ratio between two neighboring Scal-1^{hi} compartments, i.e., $r_i = n_{i\text{h}}/n_{i-1\text{h}}$, $i = 2, 3, 4$. s_i is the size ratio between Sca-1^{lo} and Sca-1^{hi} sub-compartments at each level, i.e., $s_i = n_{i\text{l}}/n_{i\text{h}}$, $i = 2, 3, 4$. The parameter ϕ is the transition rate between Sca-1^{hi} and Sca-1^{lo} subpopulations. Detailed descriptions of all parameters are given in Supplementary Table 1.

The parameter values are inferred in a standard way by minimizing the sum of the weighted squared residuals or the χ^2 value:

$$
\chi^2 = \sum_i \sum_j \left(\frac{y_{i,j}(t_j) - y_{i,j}^{(o)}(t_j)}{\sigma_{i,j}} \right)^2 \tag{26}
$$

where y represents the label frequency (normalized by ES HSC), compartment size and mean H2B-GFP intensity; i indicates different cell compartments, and j the measured time points. $\sigma_{i,j}$ is the corresponding standard error of the mean. The uncertainty of the parameters is quantified by profile likelihood method implemented by the Data2Dynamic package, where the likelihood is tightly linked to χ^2.

Model selection is done with a comprehensive array of sub-models of the most general model, thus explicitly introducing parsimony and allowing model ranking via the Akaike information criterion. The equations for any sub-model are derived from Equation 24 by setting specific parameters to zero. Moreover, we found that the experimental data cannot distinguish between cell differentiation via asymmetric cell division and direct differentiation without division (see also Barile et al., 2020). To be specific, we neglect the contribution of direct differentiation in passing on H2B-GFP label to downstream compartments, compared to label dilution via different types of cell division, and set μ_i in the corresponding equations to zero. This assumption does not compromise the quality of the fit to the data.

We fit all the models to the *Fgd5* $^{\textit{ZsGreen:CreERT2}}$ label propagation and H2B-GFP dilution data and ranked the models using the bias-corrected Akaike information criterion (AICc). The top ranked models show two common features: first, parallel differentiation via Sca-1^{hi} and Sca-1^{lo} paths; second, irreversible transition from Sca-1^{hi} to Sca-1^{lo} states. Models bearing one or neither of the two features ranked poorly.

2.2 Quantifying the probability of lineage pathways

There is one single pathway for lymphoid lineage, where Sca-1 level is consistently high from tip HSC to HPC-1. On the other hand, myeloid cells can be derived via 6 pathways. To quantify the contributions of these pathways, we calculated pathway probability based on differentiation history. We started at the

final cell state z_i, and calculated the probability of its ancestral line of cells to have differentiated via a pathway $z_0 \to z_1 \to \cdots \to z_i$. To do this, we back-tracked from state z_i and denoted the probability to have arrived from the precursor state z_{i-1} by $p(z_{i-1} \to |z_i)$. Because we consider a Markov state network, the differentiation decision at each state is independent of the differentiation history, such that the pathway probability factorizes:

$$p(z_0 \to z_1 \to \cdots z_{N-1} \to |z_N) = \prod_{i=1}^{N} p(z_{i-1} \to |z_i). \tag{27}$$

When an expanding cell population reaches a state of steady growth, the elementary transition probability can be written as:

$$\begin{aligned} p(z_{i-1} \to |z_i) &= \lim_{T\to\infty} \frac{\text{transitions from } z_{i-1} \text{ to } z_i}{\text{transitions from all precursor states to } z_i} \\ &= \lim_{T\to\infty} \frac{\int_0^T n(z_{i-1}, t)\alpha_{z_{i-1}\to z_i} dt}{\sum_z \int_0^T n(z, t)\alpha_{z\to z_i} dt} \\ &= \lim_{T\to\infty} \frac{\int_0^T e^{\Lambda t} p_{\text{sg}}(z_{i-1})\alpha_{z_{i-1}\to z_i} dt}{\sum_z \int_0^T e^{\Lambda t} p_{\text{sg}}(z)\alpha_{z\to z_i} dt} \\ &= \frac{p_{\text{sg}}(z_{i-1})\alpha_{z_{i-1}\to z_i}}{\sum_z p_{\text{sg}}(z)\alpha_{z\to z_i}} \end{aligned} \tag{28}$$

where the sums run over all possible precursor states; Λ is the dominant eigenvalue of the dynamic system, that is, the population growth rate; $p_{\text{sg}}(z_i)$ is the relative compartment size of z_i to the sum of all populations at steady growth; and α is the differentiation rate. Combining Equations 27 and 28, we calculated the probability of all six paths from tip HSC to CMP at steady growth.

2.3 Model inference including mature cell populations

We extend the current model to describe differentiation pathways of mature blood cells, with a focus on platelets. It is known that platelets arise from conventional CMP-MkP pathway. However, the labeling frequency of platelets is higher than that of MPP, which suggests that the conventional pathway is not the only route to produce platelets. We therefore extended the model to allow MkP to directly receive cells from CD201$^{-/lo}$ Sca-1^{lo} HSCs, and fit the model to the collective data of HSPC, MkP and platelets. However, the model fails to capture the label propagation data of MkP and platelets, because the measured MkP labeling frequency is still lower than platelets (Fig. 3c). A potential solution to this controversy is to assume two, in the simplest case, heterogeneous subpopulations in MkP, one of which is produced directly from HSC (Supplementary Fig. 8c). The model of heterogeneous MkP compartment is supported by the data (Supplementary Fig. 8d).

CD48$^{-/lo}$ MkP arises directly from Sca-1^{lo} HSC, while CD48^{hi} MkP is produced via the conventional CMP pathway. Moreover, the two-compartment model faithfully captured the data of MkP subpopulations.

The corresponding model equations are:

$$
\begin{aligned}
\dot{f}_{\text{mkp1}} &= \frac{\alpha_{2\text{l}\to\text{mkp1}}}{r_{\text{mkp1}}}(f_{2\text{l}} - f_{\text{mkp1}}) \\
\dot{f}_{\text{mkp2}} &= \frac{\alpha_{\text{cmp}\to\text{mkp2}}}{r_{\text{mkp2}}}(f_{\text{cmp}} - f_{\text{mkp2}}) \\
\dot{r}_{\text{mkp1}} &= \alpha_{2\text{l}\to\text{mkp1}} - \left(\kappa_{\text{mkp1}} - \kappa_{2\text{l}} + \frac{\alpha_{1\text{hl}}}{r_2 s_2} + \frac{\phi_{2\text{h}}}{s_2}\right) r_{\text{mkp1}} \\
\dot{r}_{\text{mkp2}} &= \alpha_{\text{cmp}\to\text{mkp2}} - \left(\kappa_{\text{mkp2}} - \kappa_{\text{cmp}} + \frac{\alpha_{4\text{h}\to\text{cmp}}}{r_{\text{cmp}}} + \frac{\alpha_{4\text{l}\to\text{cmp}} s_4}{r_{\text{cmp}}}\right) r_{\text{mkp2}} \\
\dot{x}_{\text{mkp1}} &= v - k_{\text{d}} x_{\text{mkp1}} + \frac{\alpha_{2\text{l}\to\text{mkp1}}}{2 r_{\text{mkp1}}} x_{2\text{l}} - \left(\frac{\lambda_{\text{mkp1}}}{2} + \frac{\alpha_{2\text{l}\to\text{mkp1}}}{r_{\text{mkp1}}}\right) x_{\text{mkp1}} \\
\dot{x}_{\text{mkp2}} &= v - k_{\text{d}} x_{\text{mkp2}} + \frac{\alpha_{\text{cmp}\to\text{mkp2}}}{2 r_{\text{mkp2}}} x_{\text{cmp}} - \left(\frac{\lambda_{\text{mkp2}}}{2} + \frac{\alpha_{\text{cmp}\to\text{mkp2}}}{r_{\text{mkp2}}}\right) x_{\text{mkp2}} \\
\dot{x}_{\text{cmp}} &= v - k_{\text{d}} x_{\text{cmp}} + \frac{\alpha_{4\text{h}\to\text{cmp}}}{2 r_{\text{cmp}}} x_{4\text{h}} + \frac{\alpha_{4\text{l}\to\text{cmp}} s_4}{2 r_{\text{cmp}}} - \left(\frac{\lambda_{\text{cmp}}}{2} + \frac{\alpha_{4\text{h}\to\text{cmp}} + \alpha_{4\text{l}\to\text{cmp}} s_4}{r_{\text{cmp}}}\right) x_{\text{cmp}}
\end{aligned}
\tag{29}
$$

where mkp1 and mkp2 in the subscripts represent CD48$^{-/\text{lo}}$ and CD48^{hi} MkP, respectively. The net cell loss of Sca-1^{lo} HSC (compartment 2l) also contains the direct differentiation path to MkP CD48$^{-/\text{lo}}$,

$$
\kappa_{2\text{l}} = (\alpha_{2\text{ll}} + \alpha_{2\text{lh}} + \alpha_{2\text{l}\to\text{mkp1}} + \mu_{2\text{ll}} + \mu_{2\text{lh}} + \mu_{2\text{l}\to\text{mkp1}} - \lambda_{2\text{l}})/2 + \phi_{2\text{l}} + \delta_{2\text{l}} \tag{30}
$$

The equations for megakaryocytes and platelets are,

$$
\begin{aligned}
\dot{f}_{\text{mk}} &= \frac{\alpha_{\text{mkp1}\to\text{mk}}}{r_{\text{mk/mkp1}}}(f_{\text{mkp1}} - f_{\text{mk}}) + \frac{\alpha_{\text{mkp2}\to\text{mk}}}{r_{\text{mk/mkp2}}}(f_{\text{mkp2}} - f_{\text{mk}}) \\
\dot{f}_{\text{plt}} &= \frac{A\alpha_{\text{mk}\to\text{plt}}}{r_{\text{plt}}}(f_{\text{mk}} - f_{\text{plt}}) \\
\dot{r}_{\text{mk/mkp1}} &= \alpha_{\text{mkp1}} + \frac{\alpha_{\text{mkp2}}}{r_{\text{mkp12}}} - \left(\alpha_{\text{mk}\to\text{plt}} - \kappa_{\text{mkp1}} + \frac{\alpha_{2\text{l}\to\text{mkp1}}}{r_{\text{mkp1}}}\right) r_{\text{mk/mkp1}} \\
\dot{r}_{\text{plt}} &= A\alpha_{\text{mk}\to\text{plt}} - \left(\delta_{\text{plt}} - \alpha_{\text{mk}\to\text{plt}} + \frac{\alpha_{\text{mkp1}\to\text{mk}}}{r_{\text{mk/mkp1}}}\right) r_{\text{plt}}
\end{aligned}
\tag{31}
$$

where

$$
r_{\text{mkp12}} \equiv \frac{n_{\text{mkp1}}}{n_{\text{mkp2}}} = \frac{r_{\text{mkp1}} s_2}{r_{\text{mkp2}} r_{\text{cmp}} r_4 r_3} \tag{32}
$$

The model assumes no proliferation for megakaryocyte, and the cytoplasm of a megakaryocyte is shed into A platelets at rate $\alpha_{\text{mk}\to\text{plt}}$.

We then use the best fit parameters to calculate the contribution of the two pathways to platelet production, which is determined by the fluxes (cell differentiated per day) via CD48$^{-/\text{lo}}$ MkP and CD48^{hi} MkP.

2.4 Rate changes upon romiplostim treatment

To quantify the effect of romiplostim on thrombopoiesis, we allowed the model parameters to change in a stepwise manner corresponding to the treatment timing. Up to 2 weeks, all parameters remain at their values for unperturbed native hematopoiesis. At this time point, we simulated romiplostim treatment by allowing a step-change of all parameters. Interestingly, the parameters along the CD48 low MkP

pathway change most compared with the unperturbed condition (Supplementary Table 1), and romiplostim preferentially regulates thrombopoiesis via CD48$^{-/lo}$ MkP pathway.

Supplementary References

1. Domen, J., Cheshier, S. H. & Weissman, I. L. The role of apoptosis in the regulation of hematopoietic stem cells: Overexpression of Bcl-2 increases both their number and repopulation potential. *J. Exp. Med.* **191**, 253–264 (2000).

2. Busch, K. *et al.* Fundamental properties of unperturbed haematopoiesis from stem cells in vivo. *Nature* **518**, 542–546 (2015).

3. Sawai, C. M. *et al.* Hematopoietic Stem Cells Are the Major Source of Multilineage Hematopoiesis in Adult Animals. *Immunity* **45**, 597–609 (2016).

4. Barile, M. *et al.* Hematopoietic stem cells self-renew symmetrically or gradually proceed to differentiation. *BioRxiv* **2020.08.06.239186**.

5. Morcos, M. N. F. *et al.* Continuous mitotic activity of primitive hematopoietic stem cells in adult mice. *J. Exp. Med.* **217** (2020).

Supplementary Table 1. Inferred parameter values for hematopoiesis at native state, and in response to romiplostim treatment and aging.

Name	Description	Best fit [95% confidence bounds] in units day^{-1}, unless specified otherwise		
		Unperturbed	Romiplostim	Aged mice
α_{1hh}	Differentiation rate of CD201^{hi}Sca-1^{hi} HSC $\rightarrow$ CD201$^{-/lo}$Sca-1^{hi} HSC	10^{-4} [2.4×10^{-5} 2×10^{-4}]	0.03 (0 0.1]	3×10^{-4} [2×10^{-4} 5×10^{-4}]
α_{1hl}	Differentiation rate of CD201^{hi}Sca-1^{hi} HSC $\rightarrow$ CD201$^{-/lo}$Sca-1^{lo} HSC	0.013 [0.01 0.015]	0.12 [0.08 0.15]	0.007 [0.005 0.01]
α_{2hh}	Differentiation rate of CD201$^{-/lo}$Sca-1^{hi} HSC $\rightarrow$ Sca-1^{hi} MPP	0.03 [0.02 0.04]	0.06 [0.02 0.13]	0.03 [0.016 0.04]
α_{2hl}	Differentiation rate of CD201$^{-/lo}$Sca-1^{hi} HSC $\rightarrow$ Sca-1^{lo} MPP	0.012 [0.008 0.018]	10^{-8} (0 0.05]	0.05 [0.35 0.08]
ϕ_{2h}	Transition rate of CD201$^{-/lo}$Sca-1^{hi} HSC $\rightarrow$ CD201$^{-/lo}$Sca-1^{lo} HSC	0.0007 (0 0.01]	0.08 [0.05 0.19]	5×10^{-8} (0 0.007]
α_{2ll}	Differentiation rate of CD201$^{-/lo}$Sca-1^{lo} HSC $\rightarrow$ Sca-1^{lo} MPP	0.001 [0.0006 0.0013]	10^{-8} (0 0.02]	5×10^{-4} [10^{-5} 0.001]
$\alpha_{2l \rightarrow mklp1}$	Differentiation rate of CD201$^{-/lo}$Sca-1^{lo} HSC $\rightarrow$ CD48$^{-/lo}$ MkP	0.05 [0.03 0.09]	0.3 [0.27 0.44]	0.03 [0.02 0.04]
α_{3hh}	Differentiation rate of Sca-1^{hi} MPP $\rightarrow$ Sca-1^{hi} HPC-1	0.008 [0.006 0.01]	0.15 [0.1 0.3]	0.01 [0.006 0.03]
α_{3hl}	Differentiation rate of Sca-1^{hi} MPP $\rightarrow$ Sca-1^{lo} HPC-1	3.5×10^{-8} (0 0.006]	0.09 (0 0.28]	2×10^{-7} (0, 0.04]
ϕ_{3h}	Transition rate of Sca-1^{hi} MPP $\rightarrow$ Sca-1^{lo} MPP	0.016 [0.013 0.017]	0.08 (0 0.14]	0.02 [0.005 0.03]
α_{3ll}	Differentiation rate of Sca-1^{lo} MPP $\rightarrow$ Sca-1^{lo} HPC-1	0.15 [0.11,0.17]	1.1 [0.5 2.0]	0.2 [0.1 0.5]

Supplementary Table 1. Inferred parameter values for hematopoiesis at native state, and in response to romiplostim treatment and aging.

Name	Description	Best fit [95% confidence bounds] in units day^{-1}, unless specified otherwise		
		Unperturbed	Romiplostim	Aged mice
$\alpha_{4h\to clp}$	Differentiation rate of Sca-1^{hi} HPC-1 $\to$ CLP	0.1 [0.08 0.15]	0.34 [0.29 0.50]	0.09 (0 0.3]
ϕ_{4h}	Transition rate of Sca-1^{hi} HPC-1 $\to$ Sca-1^{lo} HPC-1	0.04 [0.01 0.06]	10^{-8} (0 0.08]	0.04 (0 0.2]
$\alpha_{4l\to cmp}$	Differentiation rate of Sca-1^{lo} HPC-1 $\to$ CMP	0.4 [0.35 0.45]	0.6 [0.5 0.8]	4 [1.9 4)
$\alpha_{cmp\to gmp}$	Differentiation rate of CMP $\to$ GMP	0.09 [0.05 0.5]	1.1 [0.0001 4)	0.8 [0.2 4)
$\alpha_{cmp\to mep}$	Differentiation rate of CMP $\to$ MEP	0.5 [0.15 4)	3.6 [0.005 4)	3.6 [0.4 4)
$\alpha_{cmp\to mkp2}$	Differentiation rate of CMP $\to$ CD48^{hi} MkP	0.008 [0.005 0.01]	0.1 [0.08 0.12]	0.01 [0.007 0.03]
$\alpha_{mkp1\to mk}$	Differentiation rate of CD48$^{-/lo}$ MkP $\to$ Mk	0.2 [0.15 0.46]	2.2 [1.8 2.9]	0.3 [0.06 0.5]
$\alpha_{mkp2\to mk}$	Differentiation rate of CD48^{hi} MkP $\to$ Mk	0.1 [0.08 0.2]	0.22 [0.20 0.25]	0.1 (0 0.2]
$\alpha_{mk\to plt}$	Differentiation rate of Mk $\to$ platelets	4 [0.15 4)	0.5 [0.2 1.8]	0.05 [0.003 4)
λ_{1h}	Self-renewal rate of CD201^{hi}Sca-1^{hi} HSC	0.022 [0.020 0.023]	0.27 [0.26 0.28]	0.01 [0.005 0.02]
λ_{2h}	Self-renewal rate of CD201$^{-/lo}$Sca-1^{hi} HSC	0.079 [0.076 0.083]	0.3 [0.17 0.5]	0.02 (0 0.06]
λ_{2l}	Self-renewal rate of CD201$^{-/lo}$Sca-1^{lo} HSC	0.097 [0.089 0.11]	0.3 [0.18 0.5]	0.04 [0.03 0.06]
λ_{3h}	Self-renewal rate of Sca-1^{hi} MPP	0.03 [0.026 0.033]	0.2 [0.1 0.3]	10^{-7} (0 0.02]
λ_{3l}	Self-renewal rate of Sca-1^{lo} MPP	0.13 [0.12 0.15]	0.7 [0.2 1.4]	0.1 [0.04 0.5]
λ_{4h}	Self-renewal rate of Sca-1^{hi} HPC-1	0.18 [0.16 0.20]	0.3 [0.2 0.5]	0.15 [0.1 0.36]

Supplementary Table 1. Inferred parameter values for hematopoiesis at native state, and in response to romiplostim treatment and aging.

Name	Description	Best fit [95% confidence bounds] in units day^{-1}, unless specified otherwise		
		Unperturbed	Romiplostim	Aged mice
λ_{4l}	Self-renewal rate of Sca-1^{lo} HPC-1	0.32 [0.28 0.36]	0.5 [0.3 0.9]	3.7 [1.6 4)
λ_{cmp}	Self-renewal rate of CMP	0.5 [0.2 1.3]	4 [3.2 4)	3.4 (0 4)
λ_{mkp1}	Self-renewal rate of CD48$^{-/lo}$ MkP	0.1 [0.08 0.18]	1.4 [1.1 1.9]	0.2 [0.1 0.5]
λ_{mkp2}	Self-renewal rate of CD48^{hi} MkP	10^{-6} (0 0.07]	10^{-8} (0 0.1]	6×10^{-7} (0 0.18]
κ_{clp}	Net cell loss rate of CLP	0.016 [0.01 0.02]	fixed value as unperturbed	0.03 (0 4)
δ_{1h}	Death rate of CD201^{hi}Sca-1^{hi} HSC	1.7×10^{-8} (0 0.001]	fixed value as unperturbed	0.001 (0 0.004]
δ_{2h}	Death rate of CD201$^{-/lo}$Sca-1^{hi} HSC	0.03 [0.018 0.035]	fixed value as unperturbed	4×10^{-8} (0 0.005]
δ_{2l}	Death rate of CD201$^{-/lo}$Sca-1^{lo} HSC	0.03 [0.003 0.04]	fixed value as unperturbed	0.02 [0.016 0.04]
δ_{3h}	Death rate of Sca-1^{hi} MPP	2×10^{-8} (0 0.001]	fixed value as unperturbed	7×10^{-8} (0 0.008]
δ_{3l}	Death rate of Sca-1^{lo} MPP	10^{-8} (0 0.013]	fixed value as unperturbed	10^{-7} (0 0.05]
δ_{4h}	Death rate of Sca-1^{hi} HPC-1	7×10^{-8} (0 0.01]	fixed value as unperturbed	5×10^{-6} (0 0.18]
δ_{4l}	Death rate of Sca-1^{lo} HPC-1	10^{-7} (0 0.02]	fixed value as unperturbed	10^{-6} (0 0.7]
v	Basal production of H2B-GFP	0.0016 [0.0015 0.0018] (a.u. per day)	fixed value as unperturbed	fixed value as unperturbed
A	number of platelets derived from a single Mk	5.7×10^4 [2.3×10^4 10^5] (cells)	fixed value as unperturbed	fixed value as unperturbed
k_d	H2B-GFP turnover rate	$\ln(2)/42 = 0.0165$	fixed value[5]	fixed value[5]

Supplementary Table 2. Flow Cytometry Antibodies

Bone marrow HSPCs stainings

Antibody	Fluorochrome	Clone	Dilution	Isotope	Supplier/ Cat#
CD34	eF660	RAM34	1:25	Rat IgG2a, κ	eBioscience # 50-0341-80
CD34	eF450	RAM34	1:25	Rat IgG2a, κ	eBioscience # 48-0341-80
CD41	BV605	MWReg30	1:100	Rat IgG1, κ	Biolegend #133921
CD45.1	APC	A20	1:800	Mouse IgG2a, κ	Biolegend #110714
CD45.2	FITC	104	1:400	Mouse IgG2a, κ	Biolegend #109806
CD45.2	BV711	104	1:100	Mouse IgG2a, κ	Biolegend #109847
CD48	BV421	HM48-1	1:400	Ar. Hamster IgG	BD #747718
CD48	PE	HM48-1	1:400	Ar. Hamster IgG	eBioscience # 12-0481-81
CD117 (c-Kit)	APC/eF780	2B8	1:1600	Rat IgG2b, κ	eBioscience # 47-1171-80
CD127	PE/Cy7	A7R34	1:50	Rat IgG2a, κ	Biolegend #135014
CD135	BV421	A2F10	1:100	Rat IgG2a, κ	Biolegend #135315
CD150	PE/Cy7	TC15-12F12.2	1:200	Rat IgG2a, λ	Biolegend #115914
CD16/32	AF700	93	1:100	Rat IgG2a, λ	eBioscience # 56-0161-80
CD201 (EPCR)	APC	eBio1560	1:100	Rat IgG2b, κ	eBioscience # 17-2012-80
CD201 (EPCR)	PerCP/eFluor710	eBio1560	1:200	Rat IgG2b, κ	eBioscience # 46-2012-80
Ly-6A/E (Sca-1)	PCP/Cy5.5	D7	1:400	Rat IgG2a, κ	eBioscience # 45-5981-80

Peripheral blood stainings

Antibody	Fluorochrome	Clone	Dilution	Isotope	Supplier/ Cat#
CD3e	APC	145-2C11	1:100	Ar. Hs. IgG	eBioscience # 17-0031-63
CD3e	PCP/eF710	eBio500A2	1:400	Syr.Hs. IgG	eBioscience # 46-0033-80
CD11b	APC/eF780	M1/70	1:600	Rat IgG2b, κ	eBioscience # 47-0112-80
CD11b	FITC	M1/70	1:400	Rat IgG2b, κ	eBioscience # MA1-10081
CD19	PE/Cy7	eBio1D3	1:200	Rat IgG2a, κ	eBioscience # 25-0193-81
CD41	APC	eBioMWReg30	1:50	Rat / IgG1, κ	eBioscience # A16229
CD45.1	APC	A20	1:800	Mouse IgG2a, κ	Biolegend #110714
CD45.2	FITC	104	1:400	Mouse IgG2a, κ	Biolegend #109806
Ly-6C/G (Gr1)	eF450	RB6-8C5	1:1000	Rat IgG2b, k	eBioscience # 69-5931-82
Ly-6C/G (Gr-1)	PCP/Cy5.5	RB6-8C5	1:1200	Rat IgG2b, k	eBioscience # A14801
Ter119	FITC	TER-119	1:100	Rat IgG2b, k	Biolegend #116206

Biotinylated Antibodys

Antibody	Fluorochrome	Clone	Dilution	Isotope	Supplier/ Cat#
CD3e	Biotin	145-2C11	1:400	Ar. Hs. IgG	eBioscience # MA5-17657
CD4	Biotin	GK1.5	1:400	Rat IgG2b, κ	eBioscience # 13-0041-81
CD8a	Biotin	53-6.7	1:800	Rat IgG2a, κ	eBioscience # 13-0081-81
CD11b	Biotin	M1/70	1:800	Rat IgG2b, κ	eBioscience # 13-0112-81
CD19	Biotin	eBio1D3	1:400	Rat IgG2a, κ	eBioscience # 13-0193-81
CD45R (B220)	Biotin	RA3-6B2	1:400	Rat IgG2a, κ	Biolegend #103204
Ly-6C/G (Gr-1)	Biotin	RB6-8C5	1:400	Rat IgG2b, k	eBioscience # 13-5931-81
NK-1.1	Biotin	PK136	1:800	IgG2a, κ	eBioscience # 13-5941-85
Ter-119	Biotin	TER-119	1:400	Rat IgG2b, k	Biolegend #116204

Secondary Reagents

Secondary Reagent	Fluorochrome	Dilution	Supplier/ Cat#
Streptavidin	V500	1:800	BD #561419
Streptavidin	eF710	1:200	eBioscience # 49-4317-80
