# Supplementary material for: Fate mapping of hematopoietic stem cells reveals two pathways of native thrombopoiesis
Source: Nat Commun. 2022 Aug 3;13:4504. doi: 10.1038/s41467-022-31914-z (PMC9349191; doi:10.1038/s41467-022-31914-z)
Supplement: Supplementary file 3 — Reporting Summary [file 41467_2022_31914_MOESM3_ESM.pdf]

nature research

Corresponding author(s): Thomas Höfer, Alexander Gerbaulet

Last updated by author(s): Jun 24, 2022

Reporting Summary

Nature Research wishes to improve the reproducibility of the work that we publish. This form provides structure for consistency and transparency in reporting. For further information on Nature Research policies, see our Editorial Policies and the Editorial Policy Checklist.

Statistics

For all statistical analyses, confirm that the following items are present in the figure legend, table legend, main text, or Methods section.

n/a	Confirmed	
☐	☒	The exact sample size (*n*) for each experimental group/condition, given as a discrete number and unit of measurement
☐	☒	A statement on whether measurements were taken from distinct samples or whether the same sample was measured repeatedly
☐	☒	The statistical test(s) used AND whether they are one- or two-sided<br>*Only common tests should be described solely by name; describe more complex techniques in the Methods section.*
☒	☐	A description of all covariates tested
☐	☒	A description of any assumptions or corrections, such as tests of normality and adjustment for multiple comparisons
☐	☒	A full description of the statistical parameters including central tendency (e.g. means) or other basic estimates (e.g. regression coefficient) AND variation (e.g. standard deviation) or associated estimates of uncertainty (e.g. confidence intervals)
☐	☒	For null hypothesis testing, the test statistic (e.g. *F*, *t*, *r*) with confidence intervals, effect sizes, degrees of freedom and *P* value noted<br>*Give P values as exact values whenever suitable.*
☒	☐	For Bayesian analysis, information on the choice of priors and Markov chain Monte Carlo settings
☒	☐	For hierarchical and complex designs, identification of the appropriate level for tests and full reporting of outcomes
☒	☐	Estimates of effect sizes (e.g. Cohen's *d*, Pearson's *r*), indicating how they were calculated

Our web collection on statistics for biologists contains articles on many of the points above.

Software and code

Policy information about availability of computer code

Data collection	BD FACS Diva 6.1, 8.0, Miltenyi MACSquantify 2, Microsoft Excel 2016
Data analysis	MATLAB Version: 9.1.0.441655 (R2016b), The MathWorks, Inc., Natick, Massachusetts, United States.<br>R version 3.5.1 (2018-07-02), The R Foundation for Statistical Computing<br>Microsoft Excel<br>FlowJo V9.9, FlowJo V10<br>Graphpad Prism 8<br>The transcriptome analysis and modeling codes are available at Github (https://github.com/hoefer-lab/Thrombopoiesis_RNAseq and https://github.com/hoefer-lab/Thrombopoiesis_Modeling).

For manuscripts utilizing custom algorithms or software that are central to the research but not yet described in published literature, software must be made available to editors and reviewers. We strongly encourage code deposition in a community repository (e.g. GitHub). See the Nature Research guidelines for submitting code & software for further information.

Data

Policy information about availability of data

All manuscripts must include a data availability statement. This statement should provide the following information, where applicable:

- Accession codes, unique identifiers, or web links for publicly available datasets
- A list of figures that have associated raw data
- A description of any restrictions on data availability

Transcriptome data is available via NCBI GEO. Bulk RNAseq of megakaryocyte progenitor subpopulation: GSE183409; single cell RNAseq of hematopoietic stem and progenitor cells: GSE159390

Field-specific reporting

Please select the one below that is the best fit for your research. If you are not sure, read the appropriate sections before making your selection.

- [x] Life sciences
- [ ] Behavioural & social sciences
- [ ] Ecological, evolutionary & environmental sciences

For a reference copy of the document with all sections, see nature.com/documents/nr-reporting-summary-flat.pdf

Life sciences study design

All studies must disclose on these points even when the disclosure is negative.

Sample size	No a priori sample size calculations were performed. The sample size (n) of each experiment is provided in the corresponding figure legends. Sample sizes were chosen based on experience and previous publications (PMID: 32302400, PMID: 25686605, PMID: 27590115) to support meaningful and significant conclusions.
Data exclusions	Figure 1c-e, two TAM-induced Fgd5 ZsGreen:CreERT2/wt R26 LSL-tDRFP/wt mice (ie. heterozygous for the R26 Cre excision reporter) showed lower RFP labeling than Fgd5 ZsGreen:CreERT2/wt R26 LSL-tDRFP/LSL-tdRFP mice (homozygous for the R26 Cre excision reporter) and were excluded. After exclusion, all mice throughout the study were homozygous for the R26 Cre excission reporter.<br><br>In single-cell RNA-seq analysis, outlier cells in quality control metrics (small library size, low number of detected genes, high ERCC and mitochondrial reads) were excluded.<br><br>Figure 4e and Supplementary Figure 8h: 2,500 CD48hi or CD48-/lo MkPs were purified from B6.RFP donor animals and transplanted into sublethally-irradiated B6 WT recipient mice. A single recipient showed reconstitution of bone marrow LSK cells 3 weeks after transplantation, which was unexpected considering the transplanted donor cell phenotype (MkP) and most likely reflects a contamination with LSK cells. This recipient mouse was excluded from the PB analysis (Fig. 4e), but is shown in the bone marrow analysis (Supplementary Fig. 8h) .
Replication	The number of independent biological replicates and independent experiments is given in each figure legend.
Randomization	Mice assigned to different experimental groups were age- and sex-matched.
Blinding	The experimenters had to perform genotyping of the animals prior to the experiments, therefore, experimenters were not specifically blinded for genotypes and/or experimental groups. However, all mice were housed, treated and analysed under consistant and uniform conditions.

Reporting for specific materials, systems and methods

We require information from authors about some types of materials, experimental systems and methods used in many studies. Here, indicate whether each material, system or method listed is relevant to your study. If you are not sure if a list item applies to your research, read the appropriate section before selecting a response.

Materials & experimental systems

n/a	Involved in the study	
[ ]	[x]	Antibodies
[x]	[ ]	Eukaryotic cell lines
[x]	[ ]	Palaeontology and archaeology
[ ]	[x]	Animals and other organisms
[x]	[ ]	Human research participants
[x]	[ ]	Clinical data
[x]	[ ]	Dual use research of concern

Methods

n/a	Involved in the study	
[x]	[ ]	ChIP-seq
[ ]	[x]	Flow cytometry
[x]	[ ]	MRI-based neuroimaging

Antibodies

Antibodies used

For bone marrow cell surface phenotype analysis, a cocktail of biotinylated antibodies against hematopoietic lineage markers (anti-CD3e (clone 145-2C11, 1:400), anti-CD4 (clone GK1.5, 1:400), anti-CD8a (clone 53-6.7, 1:800), anti-CD11b (clone M1/70, 1:800), anti-CD19 (clone ebio1D3, 1:400), anti-LY-6C/G (Gr-1) (clone RA3-8C5, 1:400), anti-NK1.1 (clone PK136, 1:800) from eBioscience, and anti-CD45R (B220) (clone RA3-6B2, 1:400), anti-Ter119 (clone TER-119, 1:400) from Biolegend) was used, followed by a secondary staining with either 1. anti-Streptavidin (Horizon V500, 1;800, BD), anti-CD117(c-kit) (APC/eF780, clone 2B8, 1:1600, eBioscience), anti-Ly-6A/E (Sca-1) (PCP/Cy5.5, clone D7, 1:400, eBioscience), anti-CD150 (PE/Cy7, clone TC15-12F12.2, 1:200, Biolegend), anti-CD48 (BV421, clone HM48-1, 1:400, BD), anti-CD201 (EPCR) (APC, clone ebio1560, 1:100, eBioscience) and anti-CD41 (BV605, clone MWReg30, 1:100, Biolegend) for HSC and early progenitor analysis in Fgd5ZsGreen:CreERT2/wt/R26LSL-tdRFP/LSL-tdRFP -animals or 2. anti-Streptavidin (Horizon V500, 1;800, BD), anti-CD117(c-kit) (APC/eF780, clone 2B8, 1:1600, eBioscience), anti-Ly-6A/E (Sca-1) (PCP/Cy5.5, clone D7, 1:400, eBioscience), anti-CD34 (eF660, clone RAM34, 1:25, eBioscience), anti-CD127 (PE/Cy7, clone A7R34, 1:50, Biolegend), anti-CD135 (BV421, clone A2F10, 1:100, Biolegend) and anti-CD16/32 (AF700, clone 93, 1:100, eBioscience) for committed progenitor (CMP, GMP, MEP, CLP) analysis in Fgd5ZsGreen:CreERT2/wt/R26LSL-tdRFP/LSL-tdRFP-animals, or 3. anti-Streptavidin (eF710, 1;200, eBioscience), anti-CD117(c-kit) (APC/eF780, clone 2B8, 1:1600, eBioscience), anti-Ly-6A/E (Sca-1) (PCP/Cy5.5, clone D7, 1:400, eBioscience), anti-CD150 (PE/Cy7, clone TC15-12F12.2, 1:200, Biolegend), anti-CD48 (PE, clone HM48-1, 1:400, eBioscience), anti-CD201 (EPCR) (APC, clone ebio1560, 1:100, eBioscience), anti-CD34 (eF450, clone RAM34, 1:25, eBioscience) and anti-CD41 (BV605, clone MWReg30, 1:100, Biolegend) for R26rtTA/rtTA/Col1A1H2B-GFP/H2B-GFP -animals was performed.

For cell surface phenotype analysis of peripheral blood cells, cells either underwent erythrocyte lysis and were stained with anti-CD3 (APC, clone 145-2C11, 1:100, or PCP/eF710, clone eBio500A2, 1:400;), anti- CD11b (APC/eF780, clone M1/70, 1:600, or FITC, clone M1/70, 1:400), anti-CD19 (PE/Cy7, clone eBio1D3, 1;200), and anti- Ly-6C/G (Gr1) (eF450, clone RB6-8C5, 1:1000, or PCP/Cy5.5, clone RB6-8C5, 1:1200), all from eBioscience, or were stained directly with anti-CD41 (APC, clone eBioMWReg30, 1:50, eBioscience) and anti-Ter119 (FITC, clone TER-119, 1:100, Biolegend).

Peripheral blood cells of transplanted B6.CD45.1/.2 mice were stained with anti-CD3 (PCP/eF710, clone eBio500A2, 1:400;), anti-CD11b (APC/eF780, clone M1/70, 1:600), anti-CD19 (PE/Cy7, clone eBio1D3, 1;200), and anti- Ly-6C/G (Gr1) (eF450, clone RB6-8C5, 1:1000) from eBioscience and anti-CD45.1 (APC, clone A20, 1:800) and anti-CD45.2 (FITC, clone 104, 1:400) from Biolegend.

Bone marrow cells of transplanted B6.CD45.1/.2 mice were stained with anti-Streptavidin (Horizon V500, 1;800, BD), anti-CD117(c-kit) (APC/eF780, clone 2B8, 1:1600, eBioscience), anti-Ly-6A/E (Sca-1) (PCP/Cy5.5, clone D7, 1:400, eBioscience), anti-CD150 (PE/Cy7, clone TC15-12F12.2, 1:200, Biolegend), anti-CD48 (BV421, clone HM48-1, 1:400, BD), anti-CD45.1 (APC, clone A20, 1:800, Biolegend) and anti-CD45.2 (BV711, clone 104, 1:100) or with anti-Streptavidin (eF710, 1;200, eBioscience), anti-CD117(c-kit) (APC/eF780, clone 2B8, 1:1600, eBioscience), anti-Ly-6A/E (Sca-1) (PCP/Cy5.5, clone D7, 1:400, eBioscience), anti-CD150 (PE/Cy7, clone TC15-12F12.2, 1:200, Biolegend), anti-CD48 (BV421, clone HM48-1, 1:400, BD), anti-CD201/EPCR (PCP/eF710, clone ebio1560, 1:200, eBioscience), anti-CD41 (BV605, clone MWReg30, 1:100, Biolegend), anti-CD45.1 (APC, clone A20, 1:800, Biolegend) and anti-CD45.2 (FITC, clone 104, 1:400).

For more details on antibody dilutions, manufacturers, catalog and clone numbers please refer to Supplementary Table 2.

Validation

All antibodies were comercially sourced. In addition to the manufacturer`s validation, antibodies were titrated prior to use against murine tissues known to express the marker. The reported staining patterns were reproduced and appropriate titers were determined.

Animals and other organisms

Policy information about studies involving animals; ARRIVE guidelines recommended for reporting animal research

Laboratory animals

Mice (mus musculus) were used in this study. Male and female mice were used for experiments and housed in individually ventilated cages under specific-pathogen free environment (housing conditions: 12h/12h light cycle, xx°C, xx% humidity) at the Experimental Center of the Medical Faculty, TU Dresden. Fgd5ZsGreen:CreERT2/wt/R26LSL-tdRFP/LSL-tdRFP 18,19, R26rtTA/rtTA/Col1A1H2B-GFP/H2B-GFP 57 (Jax No: 016836, backcrossed for three generations to C57Bl/6J mice upon receipt), C57Bl/6JRj wt (Janvier), B6.CD45.1 (Jax No. 002014) and B6.CD45.1/.2 mice were used in this study. All experimental mice were 8-20 weeks of age at the time of induction., except for animal used in old cohorts (Figure 2i, 4i, Supplementary Figure 7), which were 50-80 weeks of age at induction.

Wild animals

No wild animals were used in this study.

Field-collected samples

No field-collected samples were used in this study.

Ethics oversight

Institutional approval was granted by the relevant authority (Landesdirektion Dresden, Ref. No. TVV91/2017, TVA 24/2017).

Note that full information on the approval of the study protocol must also be provided in the manuscript.

Flow Cytometry

Plots

Confirm that:

- [x] The axis labels state the marker and fluorochrome used (e.g. CD4-FITC).
- [x] The axis scales are clearly visible. Include numbers along axes only for bottom left plot of group (a 'group' is an analysis of identical markers).
- [x] All plots are contour plots with outliers or pseudocolor plots.
- [x] A numerical value for number of cells or percentage (with statistics) is provided.

Methodology

Sample preparation	Whole bone marrow cells were isolated by crushing long bones with mortar and pestle using PBS/2% FCS/2 mM EDTA and filtered through a 100 µm mesh. After erythrocyte lysis in hypotonic NH4Cl-buffer, cells were filtered through a 30 µm mesh. Hematopoietic lineage+ cells were removed with the lineage cell depletion kit (Miltenyi Biotec).<br>Peripheral blood was drawn into glass capillaries by retro-bulbar puncture. For identification of RFP+ platelets and erythrocytes, 1-2 µl of heparinized blood was mixed with PBS/2%FCS/2mM EDTA and incubated with monoclonal antibodies against CD41 and Ter119 (for gating see Supplementary Fig. 1c). For leukocyte analysis, erythrocyte lysis in hypotonic NH4Cl-buffer was performed twice for 5 min and cells were stained with monoclonal antibodies (Supplementary Table 2). For hemograms, blood was drawn by retro-bulbar puncture directly into EDTA-coated tubes (Sarstedt) and analyzed on a XT-2000i Vet analyzer (Sysmex).
Instrument	BD FACS Aria II, Aria II SORP, Aria III, Canto II, Miltenyi MACSquant, MACSquant 10, MACSquant YVB
Software	BD FACS Diva 6.1, BD FACS Diva 8.0, Miltenyi MACS quantify 2, FlowJo 9.9, Flow 10
Cell population abundance	The cell population abundance is given in Supplementary Figure 1 and was reported as percentage of lineage-negative bone marrow cells. For abundance and purity of sorted MkPs refer to Supplementary Figure 8f
Gating strategy	Populations were gated according to established immunophenotypes as follows: LS-K, lin- Sca-1- CD117+ cells; LSK, lin- Sca-1 + CD117+ cells; common lymphoid progenitor (CLP), lin- CD135+ CD127+; megakaryocyte progenitor (MkP), LS-K CD41+ CD150+; granulocyte-macrophage progenitor (GMP), LS-K CD16/32+ CD34+; common myeloid progenitor (CMP), LS-K CD16/32- CD34+; megakaryocyte-erythrocyte progenitor (MEP), LS-K CD16/32- CD34-; restricted hematopoietic progenitor 1 (HPC-1), LSK CD48hi CD150-; restricted hematopoietic progenitor 2 (HPC-2), LSK CD48hi CD150+; multipotent progenitor (MPP), LSK CD48-/lo CD150-; hematopoietic stem cell (HSC), LSK CD48-/lo CD150+; ES HSC, CD201hi Sca-1hi LSK CD48-/lo CD150+; CD201-/lo Sca-1hi HSC, CD201-/lo Sca-1hi LSK CD48-/lo CD150+; Sca-1hi HSC, Sca-1hi LSK CD48-/lo CD150+; CD201-/lo Sca-1lo HSC, CD201-/lo Sca-1lo LSK CD48-/lo CD150+. All gates were set with guidance by Fluorescence-Minus-One controls. See also Supplementary Figure 1.

☒ Tick this box to confirm that a figure exemplifying the gating strategy is provided in the Supplementary Information.
